# Population-level intervention and information collection in dynamic healthcare policy

**DOI:** 10.1007/s10729-017-9415-5

**Published:** 2017-09-08

**Authors:** Lauren E. Cipriano, Thomas A. Weber

**Affiliations:** 1 Ivey Business School, Western University, 1255 Western Road, London, ON N6G 0N1 Canada; 2 Ecole Polytechnique Fédérale de Lausanne, CDM-ODY 3.01, Station 5, CH-1015 Lausanne, Switzerland

**Keywords:** Medical decision making, Markov decision processes, Hepatitis C virus, Optimal stopping, Dynamic programming

## Abstract

We develop a general framework for optimal health policy design in a dynamic setting. We consider a hypothetical medical intervention for a cohort of patients where one parameter varies across cohorts with imperfectly observable linear dynamics. We seek to identify the optimal time to change the current health intervention policy and the optimal time to collect decision-relevant information. We formulate this problem as a discrete-time, infinite-horizon Markov decision process and we establish structural properties in terms of first and second-order monotonicity. We demonstrate that it is generally optimal to delay information acquisition until an effect on decisions is sufficiently likely. We apply this framework to the evaluation of hepatitis C virus (HCV) screening in the general population determining which birth cohorts to screen for HCV and when to collect information about HCV prevalence.

## Introduction

There is currently no guidance for determining the optimal schedule for collecting additional information regarding a decision to invest in a health program or technology [1, 2]. Current practice in the health decision science literature assumes that model parameters are fixed across cohorts and the value of additional information is calculated assuming the information-collection effort is initiated immediately [3–5]. However, in many cases the cost-effectiveness of a health program or technology – and, therefore, the value of additional information about one or more model parameters – may be changing over time because of trends affecting the cohort or the intervention [6]. In these cases, collecting additional information immediately may not be optimal and value-of-information calculations based on static parameter assumptions are likely to be biased. Planning over longer horizons is particularly important in health policy because, once established, clinical practice is difficult to change due to high switching costs (re-training and potentially new capital equipment expenditures), particularly if it appears that the level of service is being reduced [7].

In this paper we apply a stochastic dynamic programming approach to identify both the optimal time to change the current health intervention policy and the optimal time to collect decision-relevant information. We consider a hypothetical medical intervention for a cohort of patients. At each time, a new cohort of patients becomes eligible for the intervention and one parameter varies across the cohorts with imperfectly observable linear dynamics. We assume that the value of the intervention is linear in the dynamic parameter. In general, the (incremental) net monetary benefit of an intervention is linear in parameters with a one-time effect (e.g., the prevalence of a disease at one point in time or the outcome of a one-time screening test). When an effect accrues over time, such as for a reduction in the annual transition rate of a disease complication or death, linearity is often used as an approximation (see, e.g., [8]). At each time, the policy-maker can choose to invest in the medical intervention and/or to purchase sample information about the uncertain dynamic parameter. We demonstrate that information acquisition is best delayed until the signal is sufficiently likely to affect the optimal policy decision.

We apply this framework to the evaluation of hepatitis C virus (HCV) screening. Prior to the development of highly-effective treatments, HCV screening in the general population was not considered cost-effective [9] and universal screening was not recommended [10]. The advent of more effective therapy has changed the value of identifying infected individuals early to initiate treatment [11–15]. Recently released guidance by the Centers for Disease Control and Prevention (CDC) and the US Preventive Services Task Force (USPSTF) recommends one-time HCV screening for all individuals born between 1945 and 1965 [16, 17] although screening individuals born after 1965 may also be cost effective [13–15]. Based on our primary analysis of the National Health and Nutrition Examination Survey (NHANES), in the US general population, HCV prevalence is highest in people born around 1956 and declines thereafter at a rate of approximately 11% per birth year. Since HCV prevalence is decreasing across birth cohorts, HCV screening will only be cost-effective for a limited time or for a limited set of birth cohorts. We apply our model to simultaneously evaluate the optimal HCV-screening and information-acquisition policy.

Specifically, we apply our model to the policy decision of whether or not to perform one-time HCV screening in successive cohorts of healthy 50-year olds, who have not previously been tested for HCV, at a routine preventive health visit. Applying a traditional health economics framework, the policy-maker could decide today how many cohorts will be screened (e.g., each cohort of 50-year olds until those born in 1965 turn 50) or, to inform this decision, the policy-maker may seek additional information to be collected immediately. Our framework differs from the traditional paradigm in that each year the policy-maker makes a decision about whether to continue the one-time HCV screening program (whether or not to screen the new cohort of healthy 50-year olds) and whether to collect information about disease prevalence in this current cohort. If information is never collected, the optimal policy does not differ across frameworks. However, in our framework, the immediate decision is not limited to the decision of when to change policies, but it also includes when to collect information to inform a future change of policy. For example, the (immediately) optimal policy might be to screen each cohort of 50-year olds for the next 6 years and then collect information about HCV prevalence to inform future decision making. Delaying information acquisition until a time that the information is sufficiently likely to affect the decision increases the value of the information. In addition, from a practical perspective, collecting information years before it is likely to influence a policy change wastes immediate resources and, should something occur in the lag-time between the information-acquisition effort and the policy change, implementing the pre-determined policy change may not be optimal.

### Related literature and contribution

The relevant literature spans technology adoption, dynamic decisions in healthcare, and the value of information in healthcare.

#### **Technology adoption**

In technology-adoption models, a decision-maker considers the adoption of a technology of unknown profitability. Jensen [18] introduced a model in which information about a new technology is costlessly observed and the decision-maker can decide to adopt the new technology at any point in time. McCardle [19] presented a model in which collecting information is associated with a fixed cost; in each period the decision-maker can defer and collect information, or make a final decision to accept or reject the new technology. The optimal policy in each period is characterized by two thresholds: if the expected benefit is above the upper threshold, it is optimal to adopt the technology; if the expected benefit is below the lower threshold, it is optimal to reject the technology; and, if the expected benefit is between these two thresholds the optimal strategy is to gather information. Uncertainty about the technology’s value decreases over time and the two thresholds converge to the cost of adoption. Smith and McCardle [20] provided several meta-results, some of which we use, describing how properties of the value function of a stochastic dynamic program are preserved and propagated through finite-horizon Markov-reward and decision processes. Ulu and Smith [21] extended this work by relaxing the assumption that the decision-maker’s value of the technology can be summarized by the expected benefit, and they use more general monotone-comparative-statics techniques in terms of likelihood orders to generalize the class of signals that are observed prior to making an adoption decision.

Another line of research considered technologies, like ours, with uncertain and changing value. Rosenberg [22] found that expectation of technological improvement may delay a firm’s irreversible technology investments. Bessen [23] calculated the option value of delay for such a problem. Kornish [24] considered the choice between two uncertain technologies where each is subject to a positive network effect and explored the impact of the network effect on the optimal adoption policy. Chambers and Kouvelis [25] formulated a technology-adoption problem incorporating expected learning-curve effects.

#### **Stochastic dynamic programs in healthcare**

Sequential decisions under uncertainty are common in healthcare [26, 27]. Most healthcare applications of stochastic dynamic programs have focused on optimizing the timing of interventions for an individual patient: the decision to accept or reject an offered kidney for transplantation [28]; the optimal treatment plan for mild spheroctosis [29]; the optimal surveillance and management of ischemic heart disease [30]; the optimal time to perform a living-donor liver transplant [31, 32]; the optimal time to initiate treatment for HIV [33, 34]; the optimal timing and frequency of HCV testing from the patient perspective [35]; the optimal use of statins in patients with type 2 diabetes [36, 37]; the optimal prostate biopsy referral [38]; and, optimal cancer screening programs [39, 40]. Dynamic programming has also been applied to complex appointment scheduling problems in healthcare, including problems with patients of different clinical types/priority [41, 42]; incorporating patient no-shows [43]; and problems of sequential appointment scheduling with the objective of closely adhering to a prescribed schedule (e.g., sequential chemotherapy appointments [44]) or with the objective of satisfying patient preferences [45, 46]. Fewer examples of application to population-level policy exist. Kornish and Keeney [47] and Özaltın et al. [48] formulated the influenza-strain selection problem in a finite-horizon optimal-stopping framework. Similar to our problem, the influenza-vaccine composition decision is also an optimal-stopping problem with information acquisition; however, it has many unique characteristics that distinguish it from the problem discussed here such as an inventory deadline (finite horizon), a product useful for one season only, and a time-consuming production process. Similar to many of the technology-adoption models discussed above but unlike our framework, in the influenza-vaccine composition models, information is collected in every period in which a final decision has not yet been made.

#### **Health economics and value of information in healthcare**

Cost-effectiveness analysis is an economic method for comparing the lifetime discounted costs and health benefits associated with two or more medical interventions or health programs [1, 2]. In theory, the optimal allocation of resources across a portfolio of health interventions is determined by solving a constrained optimization problem with the objective of maximizing health benefits subject to a budget constraint [49–51]. In reality, regional and national health policy bodies routinely compare the incremental cost effectiveness ratio of candidate interventions to a pre-determined threshold intended to approximate the shadow price of the budget to determine if the intervention is ‘cost-effective’ as one component of their policy-making process [52]. Cost-effectiveness analysis is widely used to evaluate general population screening for relatively rare conditions because these programs impose a small cost on everyone who is screened and provide substantive healthcare gains for only a small number of individuals who are identified (or identified earlier than they would be otherwise); calculating the population-level costs and benefits can require detailed natural history models, extensive model calibration and validation, and thorough analysis.

Bayesian decision theory approaches to value-of-information assessment were first introduced by Raiffa and Schlaifer [53]. Weinstein [54] proposed the widespread adoption of value-of-information analysis to research priority setting in health policy and medicine. Hornberger et al. [55], Claxton and Posnett [56], and Claxton [57] introduced a Bayesian approach to identifying the optimal trial sample size and to assessing the value of additional information for technology-adoption assessments. Several approaches to increasing the accuracy of value-of-information calculations continued to relax assumptions implicit in the original formulation (see examples in [58–62]). One common assumption in these studies is that the currently estimated per-person value of information can be applied to individuals in all future cohorts. Recognizing some of the implications of this assumption, Philips et al. [6] discussed the impact that intervention-horizon uncertainty, price changes, and technological development can have on the per-person value of information for future cohorts. They find that delaying information collection may be desirable but do not provide a framework for determining the optimal time to collect information.

#### **Contribution**

In this paper, we extend the technology-adoption literature by allowing for a technology that is changing in value over time, for the opportunity to ‘wait’ without collecting information, and for the possibility of optimally determining the collected amount of information in each period. We also incorporate the possibility of an imperfect information-collection technology. We broaden the scope of applications of stochastic dynamic programs in the area of healthcare in an important way – focusing on population policy rather than patient-level decisions. We extend the health decision science literature on value-of-information assessment by developing an approach to identify the optimal information-acquisition policy when model parameters are varying across cohorts. Finally, as an example, we apply our framework to the timely public policy problem of developing a population screening program for HCV. We find that considering the opportunity to collect information in the future leads to a substantially different policy recommendation than current guidelines because it explicitly considers and addresses the parameter uncertainty which is changing over time.

## The model

A policy-maker faces recurring decisions for cohorts arriving at times *t* ∈{0,1,2,…} about whether to invest in a health intervention delivered once per cohort (of size *N*). By cohort we mean a group of individuals with a certain medical presentation (i.e., individuals with a new diagnosis of cancer) or of a certain status (i.e., individuals who turned 50 this year). The policy-maker’s objective is to maximize net monetary benefit from a societal perspective. The per-person incremental net monetary benefit (INMB) of performing the intervention compared to the *status quo* is assumed to be affine in an uncertain parameter $\tilde {p}_{t}$ that varies across the cohorts, with realizations in [0,1] and known dynamics. So $\text {INMB}_{t} = \theta \tilde {p}_{t} - \gamma $, for all *t* ≥ 0, where *𝜃* is the marginal INMB (with respect to the parameter $\tilde {p}_{t}$) and − *γ* is the fixed INMB, both measured on a per-person basis.

At the beginning of period *t*, the policy-maker simultaneously decides whether to invest in a medical intervention for the individuals in cohort *t* and whether to conduct a study of sample size *n*_*t*_ over the period to obtain a better estimate of the uncertain parameter $\tilde {p}_{t}$. Information, if sought, arrives at the end of the current period and is used, together with the known dynamics of $\tilde {p}_{t}$, to inform the intervention decision for future cohorts. Let $d_{t}\in \mathcal {D} = \{0, 1\}$ denote the intervention decision at time *t*, where *d*_*t*_ = 0 indicates *‘No intervention’* and *d*_*t*_ = 1 indicates *‘Intervention.’* The amount of information collected is measured in terms of the sample size $n_{t} \in {\mathcal N}=\{0,\ldots ,N\}$; it is obtained at the cost *K*(*n*_*t*_), where *K*(⋅) is an increasing function including a fixed and a variable cost when *n*_*t*_ > 0 and *K*(0) = 0. Thus, at each time *t* the policy-maker implements the control $u_{t}=(d_{t}, n_{t})\in \mathcal {D} \times \mathcal {N}$. The per-person current reward for the cohort in period *t* is

1 $$ g(\tilde{p}_{t}, u_{t}) = d_{t} (\theta \tilde{p}_{t} - \gamma) - \frac{K(n_{t})}{N}.  $$

The application in Section 4 features the decision problem of when to stop a once-in-a-lifetime disease-screening program where $\tilde {p}_{t}$ is the uncertain disease prevalence in the *t*-th cohort which, in expectation, is geometrically decreasing over time; *𝜃* > 0 denotes the marginal benefit of early diagnosis and treatment for an affected individual, *γ* > 0 is the per-person cost of the program, and the current-period INMB *g* is increasing in $\tilde {p}_{t}$. Beyond our leading example, the framework can accommodate a wide variety of problems. As formulated, the uncertain parameter needs to lie in a compact interval (which can be mapped via bijection to [0,1]). Thus, the parameter can represent not only a probability but also other model parameters, such as a quality-of-life weight or cost. Additionally, our analysis assumes that the parameter value is decreasing over time. To model a situation where the expectation of the uncertain parameter is increasing (e.g., obesity prevalence), the problem can be formulated as one in which a parameter of opposite definition is decreasing (e.g., prevalence of individuals who are not obese). Our exposition involves an example of when to stop a health intervention. However, the framework can also be used in situations in which the decision-maker wishes to identify the optimal time to initiate a new intervention (e.g., when to adopt a new surgical technique). More broadly, our framework can be applied in settings in which the decision-maker wishes to identify the optimal time to stop the current intervention or initiate a new intervention; the uncertain parameter is geometrically increasing or decreasing across intervention cohorts; and the current-period reward function is linearly increasing or decreasing in the uncertain parameter. Examples are shown in Table 1.

**Table 1:** Examples of alternative cases in which our framework applies

Case	Uncertain time-varying parameter $\tilde {p}_t = 1- \tilde {q}_t$	INMB_*t*_ of “Intervention” vs. “No Intervention” ^∗^	Setting ^‡^	Example ^†^
A	$\tilde {p}_t$, decreasing in *t*	INMB_*t*_ is increasing in $\tilde {p}_t$ *𝜃*,*γ* > 0	$\mu _p(x_0)>\frac {\gamma }{\theta }$ “Intervention” is currently implemented. Optimal stopping problem.	“Intervention”: General population HCV screening at age 50 (Section 4). Period reward function: $\text {INMB}_t =\theta \tilde {p}_t - \gamma $; $\tilde {p}_t$, prevalence of HCV in cohort *t*; *𝜃*, marginal benefit of early diagnosis and treatment for an infected individual; *γ*, fixed cost of screening.
B	$\tilde {p}_t$, decreasing in *t*	INMB_*t*_ is decreasing in $\tilde {p}_t$ *𝜃*,*γ* < 0	$\mu _p(x_0)>\frac {\gamma }{\theta }$ “Intervention” not currently implemented. Optimal starting problem.	“Intervention”: New surgical device vs. old device. Period reward function: $\hat {\text {INMB}}_t = -\hat {\theta } \tilde {p}_t + \hat {\gamma }$; $\tilde {p}_t$, probability of an adverse event in device iteration *t*; $\hat {\theta }$, incremental cost of an adverse event; $\hat {\gamma }$, benefit of surgical intervention without an adverse event. Problem transformation to framework: $\theta = -\hat {\theta }$, $\gamma =-\hat {\gamma }$.
C	$\tilde {q}_t$, increasing in *t*	INMB_*t*_ is decreasing in $\tilde {q}_t$ (increasing in $\tilde {p}_t$) *𝜃*,*γ* > 0	$\mu _q(x_0)<1-\frac {\gamma }{\theta }$ $\Rightarrow \mu _p(x_0)>\frac {\gamma }{\theta }$ “Intervention” is currently implemented. Optimal stopping problem.	“Intervention”: Pap smear for early identification of pre-cancerous lesions on the cervix from HPV infection. Period reward function: $\hat {\text {INMB}}_t = - \hat {\theta } \tilde {q}_t + \hat {\gamma }$; $\tilde {q}_t$, prevalence of HPV vaccine coverage in cohort *t*; $\hat {\theta }$, difference in benefit of Pap smear in a vaccinated person (compared to an unvaccinated person); $\hat {\gamma }$, value of Pap smear in an unvaccinated person. Problem transformation to framework: $\tilde {p}_t = 1-\tilde {q}_t$, $\theta = \hat {\theta } $, $\gamma =-\hat {\gamma } + \hat {\theta }$.
D	$\tilde {q}_t$, increasing in *t*	INMB_*t*_ is increasing in $\tilde {q}_t$ (decreasing in $\tilde {p}_t$) *𝜃*,*γ* < 0	$\mu _q(x_0)<1-\frac {\gamma }{\theta }$ $\Rightarrow \mu _p(x_0)>\frac {\gamma }{\theta }$ “Intervention” not currently implemented. Optimal starting problem.	“Intervention”: Peanut-free spaces regulation (in schools, airplanes, etc.). Period reward function: $\hat {\text {INMB}}_t =\hat {\theta } \tilde {q}_t - \hat {\gamma }$; $\tilde {q}_t$, prevalence of severe peanut allergy at time *t*; $\hat {\theta }$, benefit of peanut-free spaces to individuals with peanut allergies; $\hat {\gamma }$, fixed cost of creating and enforcing peanut-free public spaces. Problem transformation to framework: $\tilde {p}_t = 1-\tilde {q}_t$, $\theta = -\hat {\theta }$, $\gamma =\hat {\gamma } - \hat {\theta }$.

^*^ When *𝜃* ≤ 0 ≤ *γ*, the “Intervention” is dominated by the alternative for all realizations of $\tilde {p}_t$. For *γ* ≤ 0 ≤ *𝜃*, the “Intervention” dominates the alternative for all realizations of $\tilde {p}_t$

^‡^ *μ*_*p*_(*x*_0_) is the expectation of the initial belief $\tilde {p}_0$; *μ*_*q*_(*x*_0_) is the expectation of the initial belief $\tilde {q}_0$; *μ*_*p*_(*x*_0_) = 1 − *μ*_*q*_(*x*_0_)

^†^ In each of the examples, the period reward function is linear in the time-varying parameter. That the mean and variance of the time-varying parameter satisfy the dynamics presented in Section 2.3 should be verified empirically for each case

### The information-acquisition problem

The policy-maker’s prior belief about $\tilde {p}_{t}$ at *t* = 0 is beta-distributed with distribution parameters *x*_0_ = (*a*_0_,*b*_0_). The posterior distribution when a beta-density is updated in a Bayesian manner with information collected using an imperfect information-collection technology is a mixture of beta-densities. Thus, in general, the policy-maker’s prior beliefs about $\tilde {p}_{t}$ at time *t* are in $\mathcal {P}$ where $\mathcal {P}$ denotes the set of measures which are a mixture of beta-densities. Specifically if $\tilde {p}_{t} \in \mathcal {P}$, then there exists parameters $x_{t,i} = (a_{t,i}, b_{t,i}) \in \mathbb {R}_{++}^{2}$ for all *i* where $ 1 \leqslant i \leqslant m, m \in \mathbb {R}_{++}$, and a set of non-negative weights *ω*_*i*_ such that ${\sum }_{i=1}^{m} \omega _{i} = 1$, where the distribution of $\tilde {p}_{t}$ is a mixture of beta-densities of the form ${\sum }_{i=1}^{m} \omega _{i} \text { beta}(a_{t,i}, b_{t,i})$.

The policy-maker has the option to update his beliefs about the parameter $\tilde {p}_{t}$ by testing *n*_*t*_ individuals at cost *K*(*n*_*t*_). The information-collection technology has binary test characteristics *q* = (*q*_1_,*q*_2_), where *q*_1_ is the sensitivity, *q*_2_ is the specificity, and *q*_1_ + *q*_2_ > 1 (indicating the test is properly labeled). The terms ‘sensitivity’ and ‘specificity’ are often used to describe test accuracy in the medical literature. For clarity, we state their relationship to Type I and Type II error: ‘Specificity’ = 1 −‘Type I error’ = 1 −‘False positive rate’ and ‘Sensitivity’ = 1 −‘Type II error’ = 1 −‘False negative rate’. The number of positive samples is an uncertainty $\tilde {v_{t}}$ with realization *v*_*t*_ ∈{0,…,*n*_*t*_}. Based on the collected information the policy-maker updates his beliefs about $\tilde {p}_{t}$ in a Bayesian manner.

#### **Proposition 1**

*If the policy-maker’s prior belief* *f*_*p*_(⋅)*is a mixture of beta-densities, i.e.,* $f_{p} \in \mathcal {P}$ *,* *then for any number of positive observations* $\tilde {v}_{t} = v_{t}$ *from* *n*_*t*_ *samples, the Bayesian posterior belief* *f*_*p*|*v*_(⋅|*v*_*t*_) *is also a mixture of beta-densities, i.e.,* *f*_*p*|*v*_ *is in* $\mathcal {P}$ *.*

#### *Proof*

See Appendix A.1. □

If $\tilde {p}_{t}$ is a mixture of *m* ≥ 1 beta-densities and if the information-collection technology is imperfect (i.e., $\min \{q_{1},q_{2}\}<1$), then the true posterior distribution is also a mixture of beta-densities, containing between *m* + *n*_*t*_ and *m* × (*n*_*t*_ + 1) unique beta-distributions (see Appendix A.2.1). The resulting probability density function (pdf) is

2 $$\begin{array}{@{}rcl@{}} f_{p|v}\!(p | x_{t},\! n_{t},\!v_{t},\! q)\!\!&=&\!\! \sum\limits_{j=0}^{v_{t}} \sum\limits_{k=0}^{n_{t} -v_{t}} \sum\limits_{i=1}^{m} \omega^{\prime}_{j, k, i}\\ &&\times\text{beta}(a_{t,i}\!\,+\,j\,+\,k,\! b_{t,i}\,+\,n_{t}\!\,-\,j\,-\,k ), \end{array} $$

with updated weights

$$\omega^{\prime}_{j, k, i} = \frac{ \frac{ \omega_{i} {q_{1}^{j}} (1-q_{2})^{v_{t}-j} (1-q_{1})^{k} q_{2}^{n_{t}-v_{t}-k} {\Gamma}(a_{t,i}+b_{t,i}) {\Gamma}(a_{t,i} +j+k) {\Gamma}(b_{t,i} +n_{t}-j -k)} { {\Gamma}(j+1) {\Gamma}(v_{t}-j+1) {\Gamma}(k+1) {\Gamma}(n_{t}-v_{t}-k+1) {\Gamma}(a_{t,i}){\Gamma}(b_{t,i}) {\Gamma}(a_{t,i} + b_{t,i} +n_{t})}} { \sum\limits_{r=0}^{v_{t}} \sum\limits_{s=0}^{n_{t} - v_{t}} \sum\limits_{i=1}^{m} \frac{ \omega_{i} {q_{1}^{r}} (1-q_{2})^{v_{t}-r} (1-q_{1})^{s} q_{2}^{n_{t} - v_{t} - s} {\Gamma}(a_{t,i} + b_{t,i}) {\Gamma}(a_{t,i} +r+s) {\Gamma}(b_{t,i} +n_{t}-r -s)}{\Gamma(r+1) {\Gamma}(v_{t}-r+1) {\Gamma}(s+1) {\Gamma}(n_{t}-v_{t}-s+1){\Gamma}(a_{t,i}) {\Gamma}(b_{t,i}) {\Gamma}(a_{t,i} + b_{t,i} +n_{t})}}. $$

Explicit expressions for the conditional mean and variance, *μ*_*p*|*v*_ and *σ* *p*|*v*2, are provided in Appendix A.2.2.

#### *Remark 1*

If $\tilde {p}_{t}$ follows a mixture of *m* ≥ 1 beta-densities and the information-collection technology is perfect (i.e., *q*_1_ = *q*_2_ = 1), then the distribution of sample information, $\tilde {v}_{t}$, is a mixture of m beta-binomial distributions with the same weights *ω*_*i*_. Updating results in a posterior distribution that is a mixture of m beta-densities with pdf

3 $$\begin{array}{@{}rcl@{}} f_{p|v}(p | x_{t},\! n_{t},\! v_{t},\!q \!\!&=&\!\!(1,1))\\ \!\!&=&\!\! \sum\limits_{i=1}^{m} \omega{\prime}_{i} \text{beta}(a_{t,i}\,+\,v_{t} , b_{t,i}\,+\,n_{t}\,-\,v_{t} ), \end{array} $$

with updated weights

$$\omega^{\prime}_{i} = \frac{\omega_{i} \frac{\Gamma(a_{t,i}+b_{t,i}){\Gamma}(a_{t,i} +v_{t}){\Gamma}(b_{t,i}+n_{t}-v_{t})}{\Gamma(a_{t,i}){\Gamma}(b_{t,i}){\Gamma}(a_{t,i}+b_{t,i}+n_{t})}}{\sum\limits_{j=1}^{m} \omega_{j} \frac{\Gamma(a_{t,j}+b_{t,j}){\Gamma}(a_{t,j} +v_{t}){\Gamma}(b_{t,j}+n_{t}-v_{t})}{\Gamma(a_{t,j}){\Gamma}(b_{t,j}){\Gamma}(a_{t,j}+b_{t,j}+n_{t})}}, $$

for all *i* ∈{1,…,*m*}.

### Approximate Bayesian inference

For practically relevant sample sizes *n*_*t*_ and an imperfect information-collection technology, the number of beta-densities in the posterior distribution can become very large, thus requiring approximation. The need for distributional approximations in decision models has been recognized by Smith who proposed moment matching to replace continuous distributions by appropriate discrete ones [63]. More recently, moment-matching methods have also been used in a Markovian setting, to approximate vector-autoregressions [64]. In our Markov dynamic programming setting, we apply moment matching to approximate the exact posterior distribution which is a mixture of beta-densities with a single beta-distribution. This greatly simplifies the belief propagation compared to dealing with mixtures of beta-densities which feature an increasingly large number of coefficients with each information-collection effort and ultimately an infinite-dimensional state space.

Thus, instead of carrying forward full distribution information about the posterior mixture of beta-densities caused by an imperfect information-collection technology, the policy-maker’s posterior belief about $\tilde {p}_{t}$ is approximated by a single beta-distribution with the same mean and variance as the exact posterior distribution. The policy-maker’s prior belief is represented by the distribution parameters *x*_*t*_ = (*a*_*t*_,*b*_*t*_) and the posterior belief incorporating any information collected at time *t* is represented by the updated parameters $\hat {x}_{t} = (\hat {a}_{t}, \hat {b}_{t})$. Using the mean and variance of the exact posterior distribution, *μ*_*p*|*v*_ and *σ* *p*|*v*2, the approximate posterior belief parameters are determined using the one-to-one relationship between the standard parameters of the beta-distribution and its mean and variance^1^. We let *ψ*(*x*_*t*_,*n*_*t*_,*v*_*t*_,*q*) denote the function that generates the approximating parameters, with

$$\begin{array}{@{}rcl@{}} \hat{x}_{t} &=& \left[ \begin{array}{c} \hat{a}_{t} \\ \hat{b}_{t} \end{array} \right] \,=\, \psi(x_{t}, n_{t}, v_{t}, q)\\ &=& \left. \left(\frac{\mu_{p|v}(1\,-\,\mu_{p|v})}{\sigma^{2}_{p|v}} \,-\, 1 \right)\left[ \begin{array}{c} \mu_{p|v} \\ 1\,-\, \mu_{p|v} \end{array} \right]\right|_{(x_{t},n_{t},v_{t},q)}. \end{array} $$

In the case of a perfect information-collection technology, the preceding relations describe the policy-maker’s posterior beliefs exactly.

Mixtures of beta-distributions can be fitted to any continuous distribution on [0,1]. Thus, a single beta-distribution with the same mean and variance as a distribution formed by the mixture of beta-densities, will not always provide a satisfactory approximation. However, we focus on the special case where the time-*t* belief $\tilde {p}_{t}$ has been obtained via Bayesian updating from a single beta-prior. In this special case, approximating the mixture of beta-densities with a single beta-distribution with the same mean and variance maintains unimodality^2^ and stationarity of the state space over time.

We assessed the approximation quality using simulation in the policy-relevant region for our application (Appendix A.3). We found that the maximum distance between the cumulative density function of the exact posterior distributions and that of the approximation with matching mean and variance were generally small (< 2%), but became large when the mean was approaching zero and the standard deviation was relatively large. The quality of the approximation was very good (< 0.5*%*) when the mean was greater than 2%. We deemed the approximation to be of sufficiently high quality for our numerical analysis because our initial conditions and predicted trajectory without information acquisition rely on the regions in which the approximation is good. Also, because of relatively high fixed costs associated with information acquisition, optimal sample sizes in our numerical analysis tended to be sufficiently large that information would likely only be collected once which reduces concerns about compounding the approximation error over successive information-collection efforts.

### System dynamics

**Fig. 1:**
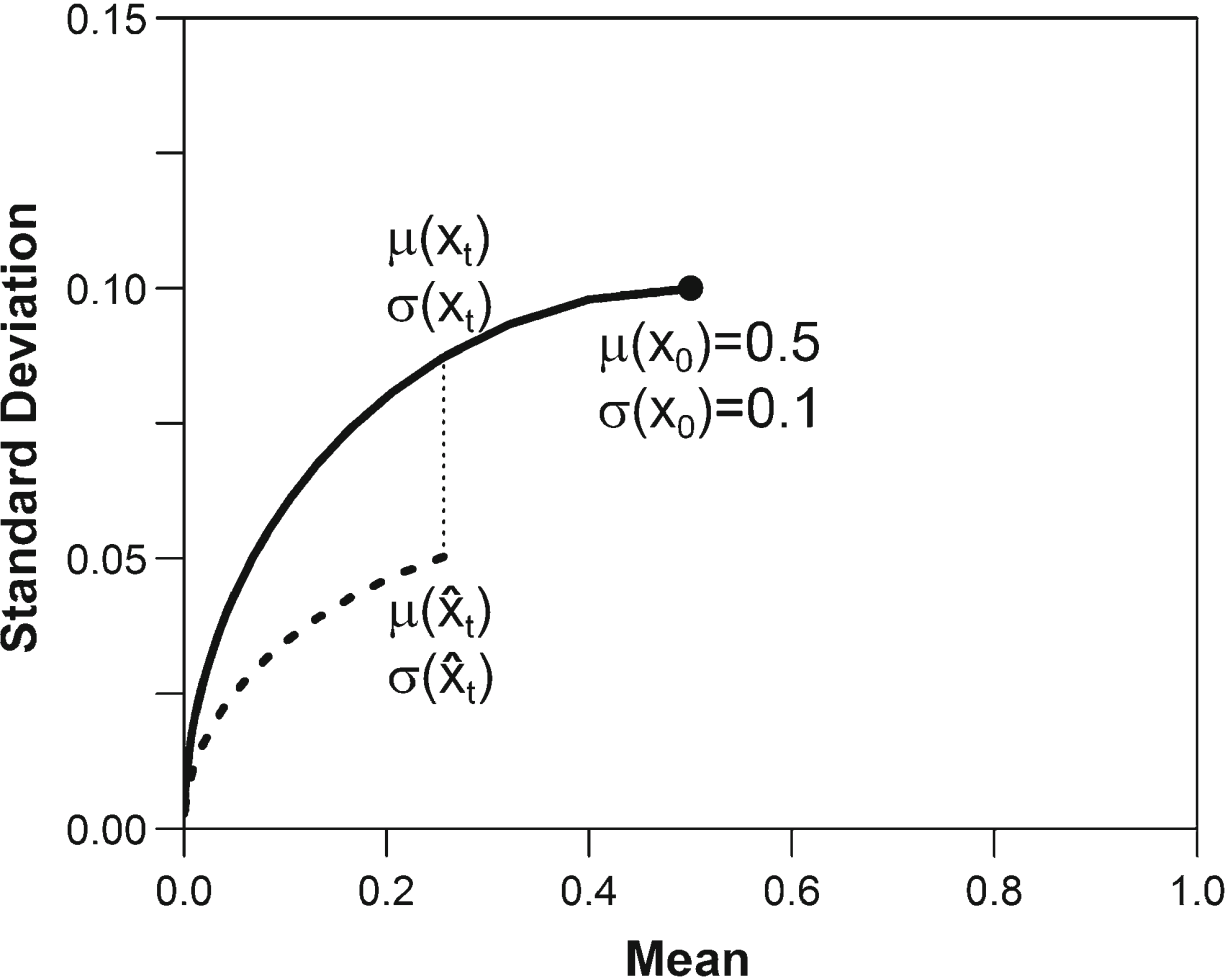
Sample state trajectory with decay *z* = 0.8, with (*dashed*) and without (*solid*) information acquisition at time *t* (for *n*_*t*_ = 50), respectively

The belief state *x*_*t*_, containing the parameters of the distribution of $\tilde {p}_{t}$, represents the policy-maker’s current beliefs about the uncertain parameter and follows a law of motion of the form

4 $$ x_{t+1} = \phi(\hat{x}_{t}) = \left[ \begin{array}{ll} z & 0\\ 1-z & 1 \end{array} \right] \hat{x}_{t} ,  $$

where *z* ∈ (0,1) is the decay rate. These dynamics imply a geometrically decreasing expected value, increasing coefficient of variation, and decreasing variance for $\mu (x_{0}) \leqslant \frac {1}{1+z}$ (Fig. 1). In the mean-variance space, the equivalent state dynamics become

$$\begin{array}{@{}rcl@{}} \mu(x_{t+1})&=& z \mu(\hat{x}_{t}) \ \ \ \text{and} \ \ \ \sigma^{2}(x_{t+1})\\ &=& \sigma^{2}(\hat{x}_{t}) \left(z + z (1-z) \left(\frac{\mu(\hat{x}_{t})}{1-\mu(\hat{x}_{t})} \right)\right). \end{array} $$

Derivations of these equations are presented in Appendix A.4. The features of these dynamics can represent a wide variety of settings in which the expectation of a parameter is geometrically decreasing over time (e.g., a health condition that is decreasing in prevalence over time; see Section 4). To model a situation where the expectation (and variance) of the uncertain parameter is increasing (e.g., obesity prevalence), the problem can be re-formulated as one in which a parameter of opposite definition is decreasing (e.g., prevalence of individuals who are not obese).

### The policy-maker’s problem

Given a social discount factor *δ* ∈ (0,1), the policy-maker’s objective is to maximize the net present value of the stream of expected INMBs, given the initial belief *x*_0_ = (*a*_0_,*b*_0_) and admissible policy decisions $U\in {\mathcal {U}} = \left \{(u_{t})_{t\in {\mathbb N}} : u_{t}=(d_{t},n_{t})\in {\mathcal D}\times {\mathcal N} \right \}$. To achieve the objective, the policy-maker seeks to find the best of all possible policies *π*_*t*_(⋅), *t* ≥ 0, with *u*_*t*_ = *π*_*t*_(*x*_*t*_) for all $x_{t}\in \mathbb {R}_{++}^{2}$, which at each time *t* maps the state space to admissible current-period actions *u*_*t*_, so that the implemented path of actions *U* = (*u*_0_,*u*_1_,…) lies in the control-constraint set $\mathcal {U}$. The number of positive observations in the testing sample of *n*_*t*_ is a random variable $\tilde {v_{t}}(n_{t})$ with realization *v*_*t*_ ∈{0,…,*n*_*t*_}. Based on the collected information the policy-maker updates his beliefs about $\tilde {p}_{t}$ in an (approximate) Bayesian manner using the function *ψ*(*x*_*t*_,*n*_*t*_,*v*_*t*_,*q*). Because of the decreasing trend of the uncertain parameter (*z* < 1), it is never optimal to restart an optimally stopped program.^3^ We consider stationary policies $\pi :\mathbb {R}_{++}^{2}\to {\mathcal D}\times {\mathcal N}$ to solve the optimal control problem

5 $$\begin{array}{@{}rcl@{}} && \max_{\pi(\cdot)} \quad \mathbb{E}\left[ {\sum}_{t=0}^{\infty} \delta^{t} \left.g(\tilde{p}_{t}, \pi(x_{t}))\right|x_{0}\right],\\ &&\text{subject to} \quad x_{t+1} = \phi(\psi(x_{t}, n_{t}, \tilde{v}_{t}(n_{t}), q)), \ \ \ x_{0} \ \ \ \text{given}, \\ && u_{t} = \pi(x_{t})\in{\mathcal D}\times{\mathcal N}. \end{array} $$

Provided the value function *V* (*x*) satisfies the Bellman equation,

6 $$\begin{array}{@{}rcl@{}} V(x) &=& \max\limits_{(d,n) \in \mathcal{D}\times{\mathcal N}} \{ d (\theta \mu(x) - \gamma ) - \frac{K(n)}{N}\\ &&\quad\quad\quad\quad\quad+ \delta \mathbb{E}[V(\phi(\psi(x, n, \tilde{v}(n),q)))\\ &&\quad\quad\quad\quad\quad\times| x, (d, n)] \}, \end{array} $$

for all admissible states $x\in \mathbb {R}_{++}^{2}$, the corresponding maximizer *π* ^∗^(*x*) on the right-hand side defines an optimal policy.

#### *Remark 2*

To reflect the policy-maker’s ongoing concern for the health-intervention decision, the problem is formulated in an infinite-horizon setting. Given a time-invariant system, this implies that the optimal policy can be described as a mapping from states to actions, without explicit consideration of time. If more information about the system becomes available over time, for example, relating to the decay rate in the system dynamics (see Eq. 4), then it is possible for the policy-maker to re-solve the problem and update the policy accordingly.

## Dynamic healthcare decisions

### Policies without information acquisition

If information is prohibitively costly or practically infeasible to collect, Eq. 6 simplifies to

$$V_{\text{NoInfo}}(x) = \max_{d \in \{0,1\}} \{ d (\theta \mu(x) - \gamma ) + \delta V_{\text{NoInfo}}(\phi(x))\}, $$

for all $x\in \mathbb {R}_{++}^{2}$, as there is no Bayesian updating and therefore *ψ* reduces to an identity map. For all states *x* for which the optimal strategy is to not do the intervention, this action remains optimal in the future because of the decreasing trend of $\tilde {p}_{t}$. Indeed, since for *z* ∈ (0,1), *μ*(*ϕ*(*x*)) = *z* *μ*(*x*) < *μ*(*x*), we have that for all states where *V* (*x*) = 0, it is also the case that *V* (*ϕ*(*x*)) = 0. Hence, for $\mu (x) \leq \frac {\gamma }{\theta }$ it is optimal to stop the intervention. This defines a threshold policy of the form

7 $$ d^{*} = \left\{ \begin{array}{ll} 0, & \text{if } \mu(x_{t}) \leq \frac{\gamma}{\theta},\\ 1, & \text{otherwise,} \end{array} \right.  $$

for all *t* ≥ 0. Restricting attention to the interesting case where $\mu (x_{0})\geq \frac {\gamma }{\theta }$ and using the fact that *μ*(*x*_*t*_) = *z* ^*t*^ *μ*(*x*_0_), we can identify the optimal time *T*(*x*_0_) to stop the intervention, which is the first period in which the intervention has a nonpositive expected INMB (see Appendix A.5):

8 $$ T(x_{0}) = \left\lceil \frac{1}{\ln(z)} \ln \left(\frac{\gamma}{\theta \mu(x_{0})} \right) \right\rceil .  $$

Hence, given any initial state *x*, the value of implementing the optimal stopping policy for *t* ∈{0,...,*T*(*x*) − 1} is

9 $$\begin{array}{@{}rcl@{}} V_{\text{NoInfo}}(x) &=& \sum\limits_{t=0}^{T(x)-1} \delta^{t}\left(\theta z^{t} \mu(x) - \gamma \right)\\ &=& \theta\mu(x) \left(\frac{1-(\delta z)^{T(x)}}{1-\delta z} \right) - \gamma \left(\frac{1-\delta^{T(x)}}{1-\delta} \right).\\ \end{array} $$

#### **Proposition 2**

*When information is prohibitively costly or practically infeasible to collect, the optimal value function* *V*_NoInfo_(*x*_*t*_) *is non-decreasing and convex in* *μ*(*x*_*t*_) *.*

#### *Proof*

See Appendix A.6. □

#### *Remark 3*

The above result depends only on the decay in the mean of the uncertain parameter distribution and is otherwise distribution-free. In other words, it does not depend on the policy-maker’s beliefs other than that $\tilde {p}_{t}$ is expected to decrease over time.

### Policies with information acquisition

When the policy-maker has the option to acquire information, the value function is determined by the Bellman equation (Eq. 6). Its properties in the no-information case (Proposition 2) carry over to the more general situation.

#### **Proposition 3**

*The optimal value function* *V* (*x*_*t*_) *is nondecreasing and convex in* *μ*(*x*_*t*_) *,* *and nondecreasing in* *σ* ^2^(*x*_*t*_) *.*

#### *Proof*

See Appendix A.7. □

#### Special case: one-time information collection

Assume for now that information can be collected at most once. Given a one-time size- *η* experiment (with *η* ≥ 1) and, briefly, ignoring the cost of information collection *K*(*η*), the value with information exceeds the no-information value,

$$\begin{array}{@{}rcl@{}} \theta \mu(x) &-& \gamma + \delta \mathbb{E}[V_{\text{NoInfo}}(\phi(\psi(x,n_{0} = \eta, \tilde{v}(\eta),q)))]\\ &-& V_{\text{NoInfo}}(x) > 0 , \end{array} $$

as a consequence of Jensen’s inequality. This insight is also useful for the comparison of experiments. A higher confidence in the information, i.e., for a larger sample size and/or better test characteristics, produces a mean-preserving spread of the random-variable $\mu (\phi (\psi (x_{t}, n_{0}=\eta , \tilde {v}_{t}(\eta ), q)))$ in the original experiment, and thus, by the convexity of the (monotone) value function and second-order stochastic dominance, a larger value with information.

Because of the monotone system dynamics, the optimal time to collect information of sample size *η*, at cost *κ*(*η*), is obtained by finding a period *k* where information acquisition is preferred to waiting until the next period, *k* + 1. In other words, find the smallest *k* for which

$$V(x_{k}|n_{k}=\eta) \geq V(x_{k+1} = \phi(x_{k})|n_{k+1}=\eta), $$

or equivalently

$$\begin{array}{@{}rcl@{}} &&\theta z^{k}\mu(x_{0}) + \frac{\delta}{1-\delta z}\\ &&\times\left(\mathbb{E}\left[V_{\text{NoInfo}}(\phi(\psi(x_{k},n_{k}=\eta,\tilde{v}(\eta),q)))]\right.\right.\\ &&-\left.\left. \delta \mathbb{E}[V_{\text{NoInfo}}(\phi(\psi(\phi(x_{k}),n_{k+1}=\eta,\tilde{v}(\eta),q)))\right] \right) \\ &\geq& \frac{1-\delta}{1-\delta z} \left(\gamma+\kappa(\eta)\right). \end{array} $$

The positivity of the right-hand side of the last inequality indicates that information acquisition may, on certain trajectories, never be optimal. This is confirmed in our application in Section 4, where the stopping region and the region with information acquisition have a common boundary, transversal to expected state trajectories.

#### General case: information collection in any period

Based on Proposition 3, the intervention is desirable for greater *μ*(*x*_*t*_) and greater *σ*(*x*_*t*_); the latter increases the upside of the policy-maker’s asymmetric (convex) payoffs, as if holding a call option. The dynamics presented in Eq. 4, with decreasing expectation and decreasing variance, imply monotonicity of the intervention decision, $d_{t+1} \leqslant d_{t}$.

##### **Corollary 1**

*Consider* *x* *t*(1)*,* *x* *t*(2)*with* *μ*(*x* *t*(1)) < *μ*(*x* *t*(2))*and* *σ* ^2^(*x* *t*(1)) = *σ* ^2^(*x* *t*(2))*,* *then if it is optimal to do the intervention with* *μ*(*x* *t*(1))*,* *it is also optimal to do the intervention with* *μ*(*x* *t*(2))*.*

##### *Proof*

See Appendix A.8. □

##### **Corollary 2**

*Consider* *x* *t*(1)*,* *x* *t*(2)*with* *μ*(*x* *t*(1)) = *μ*(*x* *t*(2))*and* *σ* ^2^(*x* *t*(1)) < *σ* ^2^(*x* *t*(2))*,* *then if it is optimal to do the intervention with* *σ*(*x* *t*(1))*,* *it is also optimal to do the intervention with* *σ*(*x* *t*(2))*.*

##### *Proof*

See Appendix A.9. □

**Fig. 2:**
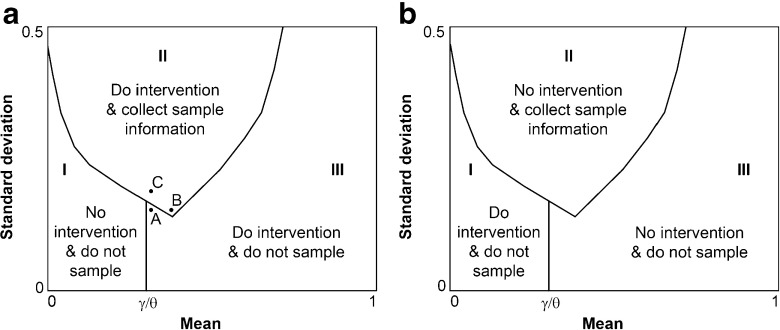
Policy regions for **a** an optimal-stopping problem and **b** an optimal-starting problem. In either case, the initial belief is in region II or III; over time, the belief moves towards region I

A direct consequence of Proposition 3 and Corollaries 1 and 2 is that an optimal policy, as a map from states to actions, features three regions (Fig. 2). We describe, in detail, the features of the optimal policy for the case of an optimal stopping problem (Fig. 2A). In region I, an optimal policy is *‘no intervention (and do not sample).’* In region II, an optimal policy is *‘do intervention and sample* *n*_*t*_ *individuals.’* In region III, an optimal policy is to *‘do intervention and do not sample.’*

The boundary between regions I and III is $\frac {\gamma }{\theta }$ (Section 3.1). For $0 \leqslant \mu (x_{t}) \leqslant \frac {\gamma }{\theta }$, the policy-maker is indifferent between *‘no intervention (and do not sample)’* and *‘do intervention and sample* *n*_*t*_ *individuals’* when the rewards of the two regions are equal:

10 $$ 0 = \theta \mu(x_{t}) - \gamma -\kappa(n_{t}) + \delta \mathbb{E}[V(\phi(\psi(x_{t},n_{t}, \tilde{v}_{t},q)))].  $$

Focusing on the region $\frac {\gamma }{\theta } \leqslant \mu (x_{t}) \leqslant 1$, the policy-maker is indifferent between *‘do intervention and sample* *n*_*t*_ *individuals’* and *‘do intervention and do not sample’* when the rewards of the two regions are equal. Removing common terms from each side, this occurs when

11 $$ - \kappa(n_{t}) + \delta \mathbb{E}[V(\phi(\psi(x_{t},n_{t}, \tilde{v}_{t}, q)))] = \delta V(\phi(x_{t})).  $$

For each *σ* ^2^(*x*_*t*_), there can exist more than one *μ*(*x*_*t*_) where $\frac {\gamma }{\theta } < \mu (x_{t}) \leqslant 1$ satisfying Eq. 11 because $V(\phi (\psi (x_{t},n_{t},\tilde {v}_{t},q)))$ is increasing, but neither concave or convex, in *v*_*t*_. The existence of the section of region III between regions I and II (the location of point A) can be obtained using intuition. Consider two points, A and B, with the same standard deviation (Fig. 2). Compared to point B, if information were to be gathered at point A, the distribution of possible posterior states includes a higher proportion of states in region I (with a reward of 0) and a lower proportion of high-reward states (those with high mean and high standard deviation) and, therefore, information acquisition is less likely to yield a value exceeding its cost. Now consider two points, A and C, with the same mean. Compared to point C, if information were to be gathered at point A, the distribution of possible posterior states is narrower. In both of these cases, increased spread on the side of low mean has no impact on the expectation and increased spread into the high-reward states substantially increases expectation. Therefore, information acquisition is more likely to yield a value exceeding its cost for the state with higher standard deviation.

##### **Proposition 4**

*For a fixed sample size* *η* *(so* *n*_*t*_ ∈{0,*η*}*for all* t*), misclassification in the information-collection technology decreases the value function and reduces the* *number of states for which information acquisition is optimal.*

##### *Proof*

See Appendix A.10. □

This result is consistent with Blackwell’s result that a less informative signal cannot increase the value of a single-person decision problem [66].

## Application

### Background and motivation

**Fig. 3:**
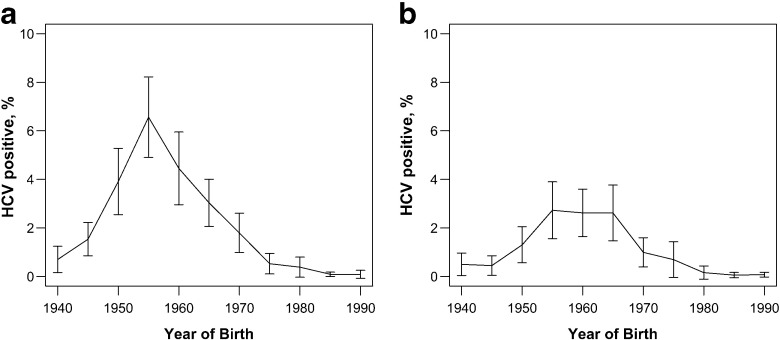
Prevalence of HCV by birth year in **a** men and **b** women. Estimated using the National Health and Nutrition Examination Survey (NHANES) (1999-2010). For details, see Appendix A.11.3

Chronic HCV infection is a slowly progressing blood-borne disease that causes liver fibrosis, cirrhosis, and liver cancer. It is the principal cause of death from liver disease and the leading indication for liver transplantation in the United States (US) [67, 68]. Between 2.7 and 5.2 million Americans (1.1% to 2.1% of the adult population) are chronically infected with HCV [69, 70]. In the non-injection drug using US population, prevalence peaks in the 1945 to 1965 birth cohorts and decreases thereafter (Fig. 3). Approximately half of all chronically infected individuals are unaware of their disease status [71].

Recent model-based analyses concluded that one-time screening of individuals born between 1945 and 1965 is cost-effective [11–15] and the CDC and USPSTF recently released new guidance in support of one-time screening of these birth cohorts [16, 17]. Several studies indicate that screening individuals born later than 1965 is also likely to be cost-effective [13–15]. Since HCV prevalence is decreasing in birth year after the 1956 birth cohort (Fig. 3), there may be a time at which screening is no longer cost-effective. To improve the decision about the best time to stop screening, additional information about prevalence of the current and future cohorts may be desirable. However, standard approaches to finding the value of information do not usually include the option to delay the information acquisition.

Note that the population we model were predominantly infected decades ago [72, 73] and do not have ongoing risk factors for HCV re-infection. Many historically significant modes of disease incidence have been virtually eliminated including transmission by surgical or other hospital equipment prior to modern sterilization procedures and blood transfusion [73, 74]. Injection equipment sharing among people who actively use injection drugs (PWID) is currently the principal cause of HCV transmission [76]. Although a history of injection drug use is relatively common among individuals with chronic HCV infection (approximately 40% [71]), re-infection and disease transmission to others via injection drug use are not an ongoing risk for a large proportion of these individuals as three-quarters of HCV infected individuals with a self-reported history of injection drug use report last injecting greater than 5 years ago (median time since last injection = 20 years) [75]. Our model does not include PWID and so we do not consider the possibility of re-infection. PWID are a high-risk population and guidelines, separate from those otherwise discussed here, recommend routine annual HCV screening in this population [77].

We now apply the stochastic dynamic programming framework developed in Section 2 to the case of one-time HCV screening at a routine medical appointment at age 50 for successive birth cohorts. We consider screening at age 50 because one-time screening at this age had the lowest incremental cost-effectiveness ratio in an analysis of single birth cohort screening [14]. Waiting to perform a one-time screening in older individuals is less cost-effective because their disease may have progressed further and treatment is less effective in more severe disease states. One-time screening of younger individuals is less cost-effective because younger individuals are further away from the long-term consequences of HCV which screening and treatment hope to avoid. We transform the unbounded state space in terms of *x*_*t*_ = (*a*_*t*_,*b*_*t*_) to the compact policy-relevant space *μ*(*x*_*t*_) and *σ*(*x*_*t*_). Using value iteration implemented in R version 2.15.0 [78], we numerically determine an optimal HCV-screening and information-collection policy for US adults.

At each time, we consider the actions of *‘do not screen for HCV and do not collect information about HCV prevalence in the current cohort;’* *‘screen for HCV and collect sample information about HCV prevalence in the current cohort;’* *‘screen for HCV and do not collect information about HCV prevalence in the current cohort.’* We compare this optimal strategy to the policies identified by various alternative approaches: a slightly modified version of the new CDC and USPSTF recommendation; an optimal policy without information acquisition; and an optimal policy with (possibly immediate) information acquisition. A policy of HCV screening does not inherently provide additional information about HCV prevalence to policy-makers, because only positive test outcomes are reported to the CDC and the reason for the medical test is private health information (the test may have been performed for a reason other than routine screening at age 50). Estimating prevalence among asymptomatic individuals seeking routine preventive medical care therefore requires a study with random sampling of those individuals. The (quasi-)linearity of INMB _*t*_ for this example is established in Appendix A.11.1. Parameter values and ranges used in sensitivity analysis are presented in Table 2. Details of parameter estimation are presented in Appendix A.11.2 and A.11.3.

**Table 2:** Model parameter values and range used in sensitivity analysis

	Males	Females	
Variable, Description	Value (Range)	Value (Range)	Sources
*Annual Cohort: Individuals aged 50 years*			
Eligible for a preventive health exam (PHE)	2.1 million (1.8 − 2.3 million)	2.1 million (1.9 − 2.4 million)	[79]
Proportion who attend a PHE	24.4% (19.3 − 29.5%)	43.3% (37.3 − 49.3%)	[80]
*N* Annual number of PHE	508, 222 (386, 600 − 630, 000)	920, 706 (753, 000 − 1, 100, 000)	Calculated
*HCV Screening Test*			
*q*_1_ Sensitivity	0.97 (0.950 − 0.999)		[81]
*q*_2_ Specificity	0.9996 (0.990 − 1.0)		[82]
*C*_*S*_ Cost of HCV-antibody test	$ 28 ($ 20 − 40)		[83]
*C*_*FP*_ Cost of false positive	$ 230 ($ 200 − 250)		[83]
*B*_*S*_ Quality-of-life change, event of screening	0 (not varied)		Assumed
*B*_*FP*_ Quality-of-life change, false-positive result	0 (not varied)		Assumed
*Lifetime discounted costs per person*			
*C*_1_ HCV+, identified through screening	$ 146, 928 ($ 140, 000 − 154, 000)	$ 161, 121 ($ 153, 000 − 170, 000)	[14]
*C*_2_ HCV+, not identified through screening	$ 126, 943 ($ 120, 000 − 133, 000)	$ 143, 900 ($ 136, 000 − 152, 000)	[14]
*C*_3_ HCV- individual	$ 181, 314 ($ 172, 250 − 190, 000)	$ 192, 135 ($ 182, 500 − 202, 000)	[14]
*Lifetime discounted quality-adjusted life-years (QALYs) per person*			
*B*_1_ HCV+, identified through screening	10.42 (10.16 − 10.68)	11.71 (11.41 − 12.0)	[14]
*B*_2_ HCV+, not identified through screening	10.06 (9.80 − 10.31)	11.44 (11.15 − 11.72)	[14]
*B*_3_ HCV- individual	15.69 (15.30 − 16.08)	16.24 (15.83 − 16.64)	[14]
*Incremental net monetary benefit (INMB) per person*			
*𝜃* Variable component of INMB	$ 7, 030 ($ 4, 000 − 12, 000)	$ 2, 962 ($ 3, 000 − 10, 000)	Eq. 20
*γ* Fixed component of INMB	$ 28.05 ($ 22 − 40)	$ 28.05 ($ 22 − 40)	Eq. 21
*Cost of collecting information*			
*K*_*F*_ Fixed cost per sampling study	$ 50, 000 ($ 25, 000 − 250, 000)		Estimated
*K*_*V*_ Variable cost per sample	$ 100 ($ 50 − 500)		[83]
*K*(*n*_*t*_) Cost of sampling	(*K*_*F*_ + *K*_*V*_ *n*_*t*_) **1**_{*n*>0}_		Assumed
*Other*			
*x*_0_ Initial belief, HCV prevalence in undiagnosed individuals	*a*_0_ = 75.1, *b*_0_ = 2350.5	*a*_0_ = 51.5, *b*_0_ = 3756.3	Appendix A.11.3
(1960 birth cohort in 2010)	*μ*(*x*_0_) = 0.031, *σ*(*x*_0_) = 0.0035	*μ*(*x*_0_) = 0.0135, *σ*(*x*_0_) = 0.0019	
*z* Rate of prevalence decay	0.893 (0.871 − 0.915)		Appendix A.11.3
*λ* Willingness-to-pay threshold	$ 75, 000/QALY gained ($ 50, 000 − 100, 000/QALY gained)		[84]
*r* Annual discount rate	0.03 (0 − 0.05)		[1]

PHE – Preventive health exam; QALY – Quality-adjusted life-year; INMB – Incremental net monetary benefit

### Results

For the purposes of our analysis, we assume the current time to be the year 2010 and the initial cohort to be born in 1960.

#### Policies identified by alternative approaches

The expected value of the CDC and USPSTF recommendation was obtained by substituting *T* = 6 into Eq. 9. The sum of the discounted expected INMBs for screening 6 cohorts at age 50, until the 1965 birth cohort turns 50 years of age, is $399.1 million for men and $15.4 million for women (Table 3). The large difference between men and women is attributable to higher HCV prevalence and higher marginal INMB of early diagnosis and treatment in men.

**Table 3:** Comparison of optimal policies indicated by various analytic approaches for men with initial belief *μ*(*x*_0_) = 0.0310 and *σ*(*x*_0_) = 0.0035 and women with initial belief *μ*(*x*_0_) = 0.0135 and *σ*(*x*_0_) = 0.0019

Case	Optimal Policy	Value (Expected INMB)	Increase in Expected INMB
Males			
CDC/USPSTF recommendation ^∗^	Screen until 1965 birth cohort turns 50	$ 399, 140, 000	Reference
No information available	Screen until 1978 birth cohort turns 50	$ 566, 470, 000	$ 167, 330, 000
Information only available immediately	Sample 910 men now, then identify optimal action	$ 566, 490, 000	$ 167, 350, 000
Information available in all periods	Sample 4,000 men in 16 years (1976 birth cohort), then identify optimal action	$ 567, 940, 000	$ 168, 800, 000
Females			
CDC/USPSTF recommendation ^∗^	Screen until 1965 birth cohort turns 50	$ 15, 390, 000	Reference
No information available	Screen until 1963 birth cohort turns 50	$ 21, 720, 000	$ 6, 330, 000
Information only available immediately	Sample 4,930 women now, then identify optimal action	$ 22, 320, 000	$ 6, 930, 000
Information available in all periods	Sample 4,500 in 1 year (1961 birth cohort), then identify optimal action	$ 22, 500, 000	$ 7, 110, 000

^*^ The new guidelines recommend screening all individuals born between 1945 and 1965 for HCV at their next routine medical visit [16, 17]. We ignore the screening of individuals born prior to 1960; for all others, we assume HCV screening occurs at age 50

We identify the threshold prevalence value below which the HCV-screening program should be terminated and the best time to terminate the screening program, assuming no opportunity to collect information using Eqs. 7–8. In men, the program should be terminated when prevalence falls below 0.4%, which will occur in 18 years (95%CI: 16-19 years). In women, the program should be terminated when prevalence falls below 0.1%, which will occur in 3 years (95%CI: 0-5 years). The expected INMB of these policies is $566.5 million for men and $21.7 million for women (Table 3).

**Fig. 4:**
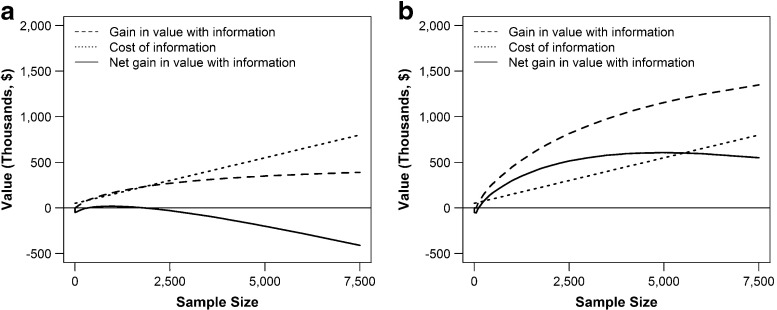
The value of collecting sample information immediately for various sample sizes. The gain in the expected INMB of the policy ($ \theta \mu (x_{0}) - \gamma + \delta \mathbb {E}[V_{\text {NoInfo}}(\phi (\psi (x_{0}, n_{0} = \eta , \tilde {v}(\eta ),q)))] $) , the cost of information (*κ*(*η*)), and the net gain in the expected INMB of an HCV-screening policy if sample information is collected in the first period only as a function of sample size for **a** men with initial belief *μ*(*x*_0_) = 0.031 and *σ*(*x*_0_) = 0.0035 and **b** women with initial belief *μ*(*x*_0_) = 0.0135 and *σ*(*x*_0_) = 0.0019

The traditional approach to value-of-information assessment in the health policy literature assumes immediate information collection [3, 4]. For men and women, we find the optimal sample sizes to be 910 and 4,930 individuals from the current cohort, respectively (Fig. 4). The expected INMB of immediate information followed by the optimal policy based on the information collected increases by $20,000 for men and $600,000 for women. Women have a greater value of immediate information because they are closer to the intervention stopping region threshold and, therefore, immediate information is more likely to result in a policy change.

#### Model results

Implementing the full model, we considered the possibility of collecting sample information at each decision period. For computational and illustrative reasons, we restricted the policy-maker’s choice to two sample sizes $\mathcal {N} \in \{0, \eta \}$. We considered several possible values for *η* (2000, 2500, 3000, ..., 8000) and we present the results for the sample size that maximized the value at the initial condition for each gender. We also performed analyses using multiple study sample size levels available at each period. We do not present these analyses, as they led to the same optimal policies indicating that our restriction to two sample sizes was not material for this application.

**Fig. 5:**
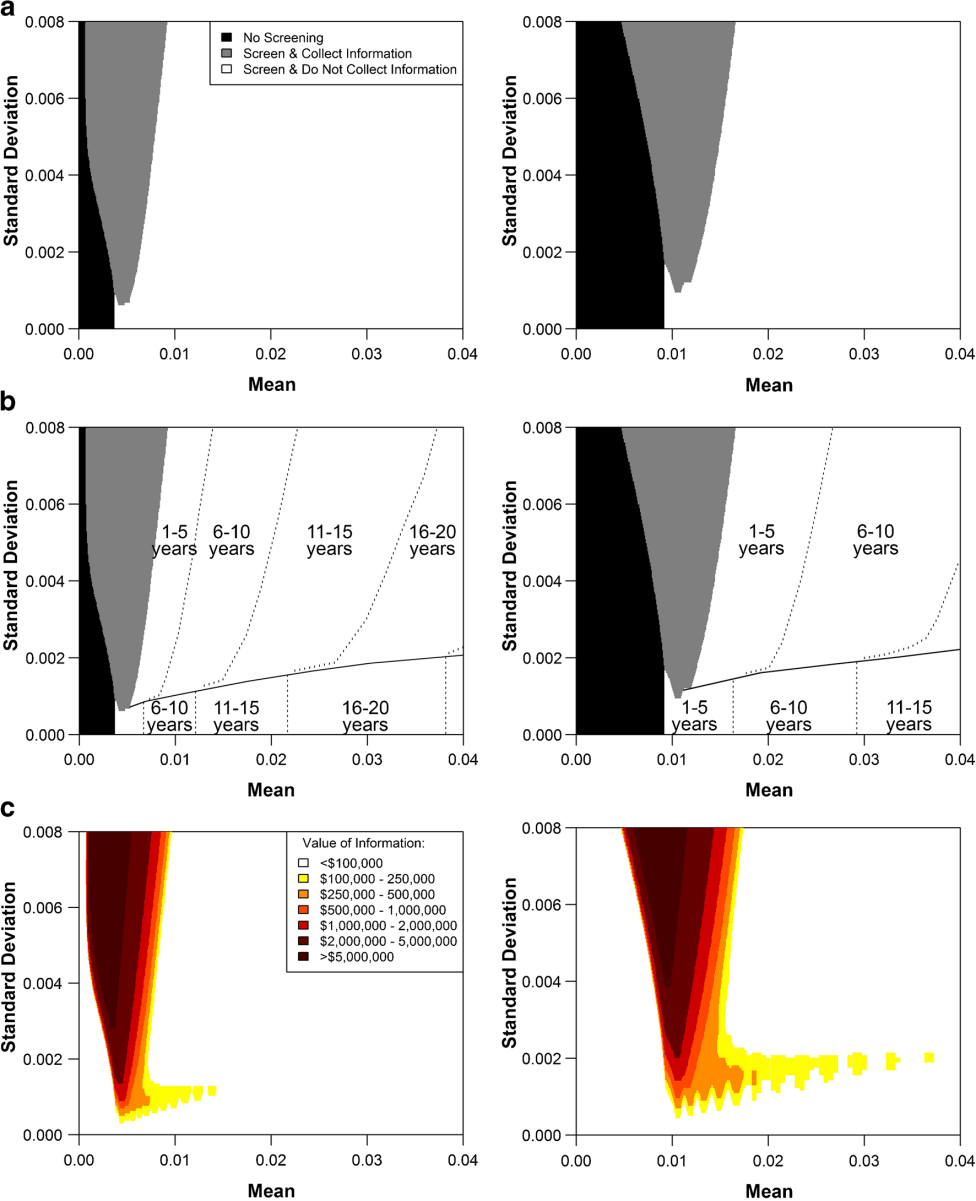
**a** Optimal policy given any current belief about HCV prevalence and the opportunity to sample 4,000 men (*left*) and 4,500 women (*right*) at any time. **b** Time to the next policy action for men (*left*) and women (*right*). For states below the solid line, the next action is to stop screening. For states above the solid line, the next action is to collect information. **c** The marginal value of collecting information, 4,000 samples for men (*left*) and 4,500 samples for women (*right*), in the current period.

The optimal policy is characterized by the three main regions described in Section 3.2.2 (Fig. 5a). At low prevalence and relatively low uncertainty, it is optimal to not screen and not collect information. At high prevalence, it is optimal to screen and not collect information. At prevalence close to the $\frac {\gamma }{\theta }$ threshold and relatively high uncertainty, it is optimal to both screen and collect information.

For each state in the region where it is optimal to screen without information acquisition, we can identify the optimal next action and the time when it should occur (Fig. 5b). We subdivide this region by a solid line. Above the solid line, which is the region with higher uncertainty, it is optimal to screen without information acquisition for a specified number of periods and then to collect information. In the region with lower uncertainty, it is optimal to screen without information acquisition for a specified number of periods and then to stop screening without ever collecting information. The current prevalence estimates for men and women indicate that it is optimal to screen without information collection for 16 years and 1 year, respectively, and then to collect sample information to inform the next action. The expected INMBs of these policies are $567.9 million and $22.5 million for men and women, respectively (Table 3).

For each state, we also computed the marginal value of collecting a specific amount of information (Fig. 5c). The marginal value of information in the current period is near-zero for states in which collecting information in the future is optimal. Consistent with our expectations, in the ‘Screen and Collect Information’ region, the marginal value of information is greatest close to the $\frac {\gamma }{\theta }$-threshold and increases with uncertainty. In the ‘Screen and Do Not Collect Information’ region, the value of information is highest along the boundary that divides the region into points with trajectories leading to information collection and points with trajectories leading to ‘No Screening’ without information collection.

Sensitivity analysis identified that the general conclusions of our numerical analysis are robust to uncertainty in the inputs (details in Appendix A.11.4).

### Discussion of application

Evaluating an HCV-screening policy over its entire lifecycle using a stochastic dynamic programming approach has led to several important policy-relevant insights. Our analysis indicates that recommendations by the CDC and USPSTF to screen individuals born between 1945 and 1965 at their next routine medical visit are conservative for men. Specifically, our analysis shows that, for men, screening should continue until at least the 1976 birth cohort turns 50 (in 2026), at which point 4,000 individuals should be sampled to inform about the continuation of the program. Screening men at least 10 years longer will enable early diagnosis in an estimated 50,500 additional individuals, thus preventing an expected 767 additional liver cancers and about 212 additional liver transplants. For women, we find that a large information-acquisition effort should take place when the 1961 birth cohort turns 50 (in 2011),^4^ as it is likely not cost-effective to screen women, per guidance, to the 1965 cohort because of relatively low prevalence (Fig. 3) and slower disease progression in women [85]. Compared to the CDC and USPSTF recommendation, our model increases the expected INMB by $168.8 million in men and $7.1 million in women.

Our analysis has several limitations. First, we assume only the current cohort can be sampled to learn about subsequent cohorts, relying on the correlation between cohorts (as implied by the system dynamics). In practice, for our example, it is possible to sample the next cohort (49-year olds) directly. We chose this assumption because the individuals who make up the ‘next cohort’ are typically unknown (e.g., the next cohort of patients with a heart attack, the next cohort of pregnant women, or the next cohort of cancer patients). Second, we consider one-time screening at age 50 based on a cost effectiveness analysis of once-in-a-lifetime HCV screening [14]. However, this analysis (and, consequently, ours) assumed that the cohort being screened has not been previously screened. Our model does not identify the optimal age at which to perform one-time screening. Third, we assumed that the individuals who attend a preventive health exam and participate in recommended HCV screening are an unbiased sample from the cohort–that is, individuals are not more or less likely to attend their preventive health exam if they are HCV-positive. However, if individuals at higher-risk of HCV disproportionately self-select for general population screening, then we have underestimated the duration for which screening will be cost-effective. If individuals at lower-risk disproportionately self-select for screening (often called the “worried well”), then we have overestimated the duration for which screening will be cost-effective. Fourth, we focus on HCV screening policy in the non-injection drug using population only because they were the focus of the recent change in HCV screening policy. Finally, while uncertainty (and related information acquisition) with respect to model parameters other than prevalence can be treated in an analogous manner, the details are left for future work.

## Conclusion

Our analysis shows that when parameters vary across intervention cohorts, it may be optimal to delay information acquisition. This is a significant improvement over the current paradigm which only considers one-time immediate information collection. More specifically, we provide a framework for optimal information acquisition, in terms of timing and precision of the acquired signal (sample size). Further, we incorporate misclassification from an imperfect information-collection technology into our framework, which is an important real-life complexity of information gathering that adds substantial analytical difficulty.

The common assumption that the per-person value-of-information remains constant for future cohorts may result in a significant error when estimating the population value of additional information. It may indicate immediate expensive information collection when, incorporating the system dynamics, the optimal action is to collect information in the future or never at all. When a parameter is evolving across intervention cohorts, ignoring the opportunity to wait and collect information in the future, when the information collected is more likely to result in action, is a missed opportunity for increased efficiency. As seen in our example, adding the option of delaying information acquisition until a time when the signal is more likely to justify a policy shift can increase the expected value compared to a policy of immediate information collection. The dynamic programming framework developed in this paper enables an accurate assessment of the marginal value of additional information and identifies an optimal information-acquisition policy.

In this work, we assumed that the dynamics are monotonically increasing or decreasing and that they are deterministic. In future work, we plan to consider the more realistic assumption of uncertainty in the dynamics. This would then enable learning about the evolution of the parameters, rather than just their current state. Furthermore, our model does not consider the possibility of intervening on a cohort at a different time in the course of their disease or lives (i.e., at an earlier or later age) or the possibility of the intervention modifying the population-level dynamics. Although true for our application, this latter assumption does not hold in general for an infectious disease. Including the additional benefits of reduced disease transmission from prevention and treatment interventions may generate more near-term benefits and may dramatically alter the value of the intervention over time.

With strained resources for health programs and population-health monitoring, this type of analysis may ensure an optimal implementation horizon for health programs together with guidance on when and how much information should be collected to inform health-program adjustments. Beyond health, many application areas face limited resources for investment and information acquisition, high-quality decision-relevant information is often difficult or expensive to collect, and population or environmental trends influence the preferences and behavior of customers across industries. Facing a dynamic consumer, competitive, or physical environment, the optimal timing of high-quality information acquisition may provide competitive advantage.

## Figures and Tables

**Fig. 6:**
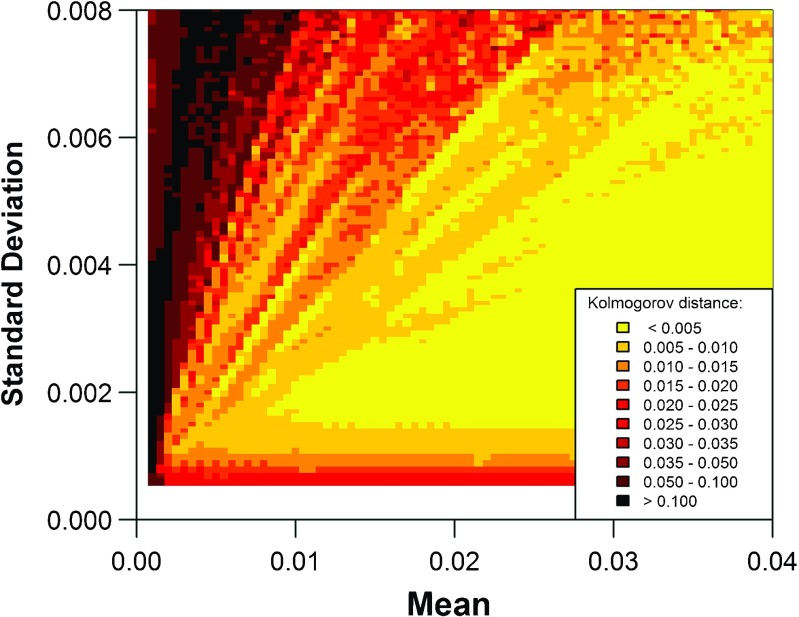
Average Kolmogorov-distance between an exact beta-mixture distribution and a single beta-distribution with the same mean and standard deviation

**Fig. 7:**
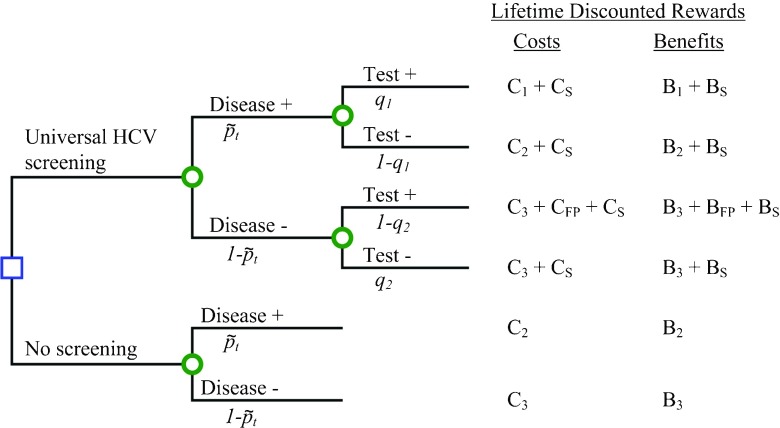
A schematic of the medical intervention decision. Each year, the policy-maker must choose whether or not to screen for HCV infection in 50-year olds who attend a routine preventive health exam

## Appendix

A glossary of symbols is provided in Table 4.

**Table 4:** Glossary of Symbols

Symbol	Definition
*N*	Annual number of preventive health exams
*HCV Screening Test*	
*q*_1_	Sensitivity
*q*_2_	Specificity
*C*_*S*_	Cost of HCV-antibody test
*C*_*F**P*_	Cost of false positive
*B*_*S*_	Quality-of-life change, event of screening
*B*_*F**P*_	Quality-of-life change, false-positive result
*Lifetime discounted costs per person*	
*C*_1_	HCV+, identified through screening
*C*_2_	HCV+, not identified through screening
*C*_3_	HCV- individual
*Lifetime discounted quality-adjusted life-years (QALYs) per person*	
*B*_1_	HCV+, identified through screening
*B*_2_	HCV+, not identified through screening
*B*_3_	HCV- individual
*Incremental net monetary benefit (INMB) per person*	
*𝜃*	Variable component of INMB
*γ*	Fixed component of INMB
*Cost of collecting information*	
*K*_*F*_	Fixed cost per sampling study
*K*_*V*_	Variable cost per sample
*K*(*n*_*t*_)	Cost of sampling
*Other*	
*x*_0_	Initial belief, HCV prevalence in undiagnosed individuals (1960 birth cohort in 2010)
*z*	Rate of prevalence decay
*λ*	Willingness-to-pay threshold
*r*	Annual discount rate

### A.1 Proof of Proposition 1

The special case where $\tilde {p}_{t}$ is beta-distributed with parameters *x*_*t*_ = (*a*_*t*_,*b*_*t*_) and the information-collection technology is perfect, i.e., where *q* = (1, 1), is well known [53]. The sample information $\tilde {v_{t}}$, where $\tilde {v_{t}}$ is the number of observed positives of *n*_*t*_ samples, is beta-binomially distributed, and updating the prior with the sample information results in a beta-distributed posterior belief with parameters (*a*_*t*_ + *v*_*t*_,*b*_*t*_ + *n*_*t*_ − *v*_*t*_).

Consider the interesting case with a prior belief *f*_*p*_(*x*_*t*_) which is a mixture of *m*_*t*_ ≥ 1 beta-distributions where *x*_*t*_ = (*x*_*t*,1_,*x*_*t*,2_,...,*x*_*t*,*m*__*t*_) and $x_{t,i} = (a_{t,i}, b_{t,i}) \in \mathbb {R}_{++}^{2}$ such that $ 1 \leqslant i \leqslant m$, and a set of positive weights *ω*_*i*_ such that ${\sum }_{i=1}^{m} \omega _{i} = 1$. The pdf is

$$f_{p}(p|x_{t}) = \sum\limits_{i=1}^{m} w_{i} \frac{\Gamma(a_{i} + b_{i})}{\Gamma(a_{i}) {\Gamma}(b_{i})} p^{a_{i} -1} (1-p)^{b_{i} -1} . $$

At time *t* the policy-maker chooses *n*_*t*_ > 0, indicating that they will collect *n*_*t*_ Bernoulli trials. The information-collection technology, with test sensitivity *q*_1_ and test specificity *q*_2_, is imperfect. The probability of observing a ‘positive’ signal from any single Bernoulli trial is $\tilde {p}_{t} q_{1} + (1-\tilde {p}_{t})(1-q_{2})$. Therefore,

$$\begin{array}{@{}rcl@{}} f_{v|p}(v_{t}|\tilde{p}_{t} \,=\, p, n_{t}, q) \!&=&\! {n_{t} \choose v_{t}} [p q_{1} \,+\, (1\,-\,p)(1\,-\,q_{2})]^{v_{t}}\\ &&\times [1 \,-\, p q_{1}\,-\, (1\,-\,p)(1\,-\,q_{2})]^{n_{t} - v_{t}}. \end{array} $$

The resulting distribution of sample information is effectively a weighted beta-binomial distribution, correcting for the additional uncertainty introduced by the imperfect information-collection technology:

12 $$\begin{array}{@{}rcl@{}} f_{v}(v_{t} | x_{t}, n_{t}, q) \!\!&=&\!\! {{\int}_{0}^{1}} f_{v|p}(v_{t}|\tilde{p}_{t} \,=\, p_{t}, n_{t}, q) f_{p}(\tilde{p}_{t}\,=\, p_{t} | x_{t}) dp \\ \!\!&=&\!\! {{\int}_{0}^{1}} {n_{t} \choose v_{t}} [p q_{1} \,+\, (1\,-\,p)(1\,-\,q_{2})]^{v_{t}} [1 \,-\, p q_{1} \,-\, (1\,-\,p)(1\,-\,q_{2})]^{n_{t} - v_{t}} f_{p}(\tilde{p}_{t}\,=\, p_{t} | x_{t}) dp \\ \!\!&=&\!\! {{\int}_{0}^{1}} {n_{t} \choose v_{t}} [p q_{1} \,+\, (1\,-\,p)(1\,-\,q_{2})]^{v_{t}} [p(1\,-\, q_{1}) \,+\, (1\,-\,p)q_{2}]^{n_{t} - v_{t}} \!\times\! \sum\limits_{i=1}^{m} \omega_{i} \frac{\Gamma(a_{t,i}\,+\,b_{t,i})}{\Gamma(a_{t,i}){\Gamma}(b_{t,i})} p^{a_{t,i} -1} (1\,-\,p)^{b_{t,i}-1} dp \\ \!\!&=&\!\! {{\int}_{0}^{1}} {n_{t} \choose v_{t}} \left(\sum\limits_{j=0}^{v_{t}} {v_{t}\choose j} p^{j} {q_{1}^{j}} (1\,-\,p)^{v_{t}-j} (1\,-\,q_{2})^{v_{t}-j} \right) \left(\sum\limits_{k=0}^{n_{t}-v_{t}} {n_{t}-v_{t}\choose k} p^{k} (1\,-\,q_{1})^{k} (1\,-\,p)^{n_{t}-v_{t}-k} q_{2}^{n_{t}-v_{t}-k} \right) \\ &&\!\!\times \sum\limits_{i=1}^{m} \omega_{i} \frac{\Gamma(a_{t,i}\,+\,b_{t,i})}{\Gamma(a_{t,i}){\Gamma}(b_{t,i})} p^{a_{t,i} -1} (1\,-\,p)^{b_{t,i}-1} dp \\ \!\!&=&\!\!\sum\limits_{j=0}^{v_{t}} \sum\limits_{k=0}^{n_{t}-v_{t}} {n_{t} \choose v_{t}} {v_{t}\choose j} {n_{t}-v_{t}\choose k} {q_{1}^{j}} (1\,-\,q_{2})^{v_{t}-j} (1\,-\,q_{1})^{k} q_{2}^{n_{t}-v_{t}-k} \!\times\!\sum\limits_{i=1}^{m} \omega_{i} \frac{\Gamma(a_{t,i}\,+\,b_{t,i})}{\Gamma(a_{t,i}){\Gamma}(b_{t,i})} {{\int}_{0}^{1}} p^{a_{t,i}+j+k -1} (1\,-\,p)^{b_{t,i}+n_{t}-j-k-1} dp \\ \!\!&=&\!\!\sum\limits_{j=0}^{v_{t}} \sum\limits_{k=0}^{n_{t}-v_{t}} {n_{t} \choose v_{t}} {v_{t}\choose j} {n_{t}-v_{t}\choose k} {q_{1}^{j}} (1\,-\,q_{2})^{v_{t}-j} (1\,-\,q_{1})^{k} q_{2}^{n_{t}-v_{t}-k} \!\times\!\sum\limits_{i=1}^{m} \omega_{i} \frac{\Gamma(a_{t,i}\,+\,b_{t,i})}{\Gamma(a_{t,i}){\Gamma}(b_{t,i})} \frac{\Gamma(a_{t,i}\,+\,j\,+\,k){\Gamma}(b_{t,i}\,+\,n_{t}\,-\,j\,-\,k)}{\Gamma(a_{t,i}\,+\,b_{t,i}\,+\,n_{t})} \\ \!\!&=&\!\! \sum\limits_{j=0}^{v_{t}} \sum\limits_{k=0}^{n_{t} -v_{t}} {q_{1}^{j}} (1\,-\,q_{2})^{v_{t}-j} (1\,-\,q_{1})^{k} q_{2}^{n_{t} - v_{t}-k}\!\times\!\sum\limits_{i=1}^{m} \omega_{i} \frac{\Gamma(n_{t}\,+\,1){\Gamma}(a_{t,i} \,+\, b_{t,i}) {\Gamma}(a_{t,i} \,+\,j\,+\,k) {\Gamma}(b_{t,i} \,+\,n_{t}\,-\,j \,-\,k)}{\Gamma(j\,+\,1) {\Gamma}(v_{t}\,-\,j\,+\,1) {\Gamma}(k\,+\,1) {\Gamma}(n_{t}\,-\,v_{t}\,-\,k\,+\,1){\Gamma}(a_{t,i}) {\Gamma}(b_{t,i}) {\Gamma}(a_{t,i} \,+\, b_{t,i} \,+\,n_{t})}. \\ \end{array} $$

Using Bayes’ Theorem, the posterior distribution is a mixture of beta-distributions with weights summing to one:

13 $$\begin{array}{@{}rcl@{}} f_{p|v}(p | x_{t}, n_{t}, v_{t}, q) \!\!&=&\!\! \frac{f_{v|p}(\tilde{v}_{t}\,=\,v_{t} | \tilde{p}, n_{t}, q) f_{p}(\tilde{p}_{t} \,=\, p_{t} | x_{t})}{f_{v}(\tilde{v}_{t}\,=\,v_{t}| x_{t}, n_{t}, q)} \\ \!\!&=&\!\! \frac{{n_{t} \choose v_{t}} [p q_{1} \,+\, (1\,-\,p)(1\,-\,q_{2})]^{v_{t}} [p(1\,-\, q_{1}) \,+\, (1\,-\,p)q_{2}]^{n_{t} - v_{t}} {\sum}_{i=1}^{m} \omega_{i} \frac{\Gamma(a_{t,i}+b_{t,i})}{\Gamma(a_{t,i}){\Gamma}(b_{t,i})} p^{a_{t,i} -1} (1\,-\,p)^{b_{t,i}-1}} {f_{v}(\tilde{v}_{t}\,=\,v_{t}| x_{t}, n_{t}, q)} \\ \!\!&=&\!\!\frac{1}{f_{v}(\tilde{v}_{t}\,=\,v_{t}| x_{t}, n_{t}, q)} {n_{t} \choose v_{t}} \left(\sum\limits_{j=0}^{v_{t}} {v_{t}\choose j} p^{j} {q_{1}^{j}} (1\,-\,p)^{v_{t}-j} (1\,-\,q_{2})^{v_{t}-j} \right) \\ & &\!\!\times \left(\sum\limits_{k=0}^{n_{t}-v_{t}} {n_{t}-v_{t}\choose k} p^{k} (1\,-\,q_{1})^{k} (1\,-\,p)^{n_{t}-v_{t}-k} q_{2}^{n_{t}-v_{t}-k} \right) \sum\limits_{i=1}^{m} \omega_{i} \frac{\Gamma(a_{t,i}\,+\,b_{t,i})}{\Gamma(a_{t,i}){\Gamma}(b_{t,i})} p^{a_{t,i} -1} (1\,-\,p)^{b_{t,i}-1} \\ \!\!&=&\!\!\frac{1}{f_{v}(\tilde{v}_{t}\,=\,v_{t}| x_{t}, n_{t}, q)} \sum\limits_{j=0}^{v_{t}} \sum\limits_{k=0}^{n_{t}-v_{t}} {n_{t} \choose v_{t}} {v_{t}\choose j} {n_{t}\,-\,v_{t}\choose k} {q_{1}^{j}} (1\,-\,q_{2})^{v_{t}-j} (1\,-\,q_{1})^{k} q_{2}^{n_{t}-v_{t}-k} \\ & &\!\!\times \sum\limits_{i=1}^{m} \omega_{i} \frac{\Gamma(a_{t,i}\,+\,b_{t,i})}{\Gamma(a_{t,i}){\Gamma}(b_{t,i})} p^{a_{t,i}+j+k -1} (1\,-\,p)^{b_{t,i}+n_{t}-j-k-1} \\ \!\!&=&\!\!\frac{1}{f_{v}(\tilde{v}_{t}\,=\,v_{t}| x_{t}, n_{t}, q)} \sum\limits_{j=0}^{v_{t}} \sum\limits_{k=0}^{n_{t}-v_{t}} \sum\limits_{i=1}^{m} {q_{1}^{j}} (1\,-\,q_{2})^{v_{t}-j} (1\,-\,q_{1})^{k} q_{2}^{n_{t}-v_{t}-k} \omega_{i} \\ & &\!\!\times \frac{\Gamma(n_{t}\,+\,1) {\Gamma}(a_{t,i}\,+\,b_{t,i}) {\Gamma}(a_{t,i} \,+\,j\,+\,k) {\Gamma}(b_{t,i} \,+\,n_{t}\,-\,j \,-\,k)} { {\Gamma}(j\,+\,1) {\Gamma}(v_{t}\,-\,j\,+\,1) {\Gamma}(k\,+\,1) {\Gamma}(n_{t}\,-\,v_{t}\,-\,k\,+\,1) {\Gamma}(a_{t,i}){\Gamma}(b_{t,i}) {\Gamma}(a_{t,i} \,+\, b_{t,i} \,+\,n_{t})} \!\times\!\text{beta}(a_{t,i}\,+\,j\,+\,k , b_{t,i}\,+\,n_{t}\,-\,j\,-\,k ) \\ \!\!&=&\!\! \sum\limits_{j=0}^{v_{t}} \sum\limits_{k=0}^{n_{t} -v_{t}} \sum\limits_{i=1}^{m} \omega^{\prime}_{j, k, i} \text{beta}(a_{t,i}\,+\,j\,+\,k , b_{t,i}\,+\,n_{t}\,-\,j\,-\,k ), \end{array} $$

with updated weights

$$\omega^{\prime}_{j, k, i} = \frac{ \frac{ \omega_{i} {q_{1}^{j}} (1-q_{2})^{v_{t}-j} (1-q_{1})^{k} q_{2}^{n_{t}-v_{t}-k} {\Gamma}(a_{t,i}+b_{t,i}) {\Gamma}(a_{t,i} +j+k) {\Gamma}(b_{t,i} +n_{t}-j -k)} { {\Gamma}(j+1) {\Gamma}(v_{t}-j+1) {\Gamma}(k+1) {\Gamma}(n_{t}-v_{t}-k+1) {\Gamma}(a_{t,i}){\Gamma}(b_{t,i}) {\Gamma}(a_{t,i} + b_{t,i} +n_{t})}} { \sum\limits_{r=0}^{v_{t}} \sum\limits_{s=0}^{n_{t} - v_{t}} \sum\limits_{i=1}^{m} \frac{ \omega_{i} {q_{1}^{r}} (1-q_{2})^{v_{t}-r} (1-q_{1})^{s} q_{2}^{n_{t} - v_{t} - s} {\Gamma}(a_{t,i} + b_{t,i}) {\Gamma}(a_{t,i} +r+s) {\Gamma}(b_{t,i} +n_{t}-r -s)}{\Gamma(r+1) {\Gamma}(v_{t}-r+1) {\Gamma}(s+1) {\Gamma}(n_{t}-v_{t}-s+1){\Gamma}(a_{t,i}) {\Gamma}(b_{t,i}) {\Gamma}(a_{t,i} + b_{t,i} +n_{t})}}. $$

The coefficients of each component of the mixture distribution sum to 1 and, therefore, the posterior distribution is a mixture of beta-distributions, which concludes our proof.

### A.2 General properties of mixtures of beta-distributions

#### A.2.1 The number of component distributions in the posterior mixture distribution

##### **Perfect information-collection technology** (i.e., *q*_1_ = *q*_2_ = 1).

If $\tilde {p}_{t}$ is a mixture of *m* ≥ 1 beta-distributions with weights *ω*_*i*_ and the information-collection technology is perfect, the distribution of sample information is a mixture of beta-binomial distributions with weights *ω*_*i*_. Updating results in a posterior distribution that is a mixture of *m* beta-distributions with parameters (*a*_*t*,*i*_ + *v*_*t*_,*b*_*t*,*i*_ + *n*_*t*_ − *v*_*t*_).

Consider the example where *m* = 2, *x*_*t*_ = ((3, 7), (7, 3)), *ω* = (0.8, 0.2), *n*_*t*_ = 10, and *v*_*t*_ = 4. Let $\hat {x}_{t}$ denote the posterior belief state. Then, $\hat {x}_{t}=((3+4,7+6), (7+4,3+6))=((7,13), (11,9))$ and *ω* ^′^ = (0.8, 0.2).

##### **Imperfect information-collection technology** (i.e., $\min \{q_{1},$*q*_2_} < 1).

If $\tilde {p}_{t}$ is a mixture of *m* ≥ 1 beta-distributions with weights *ω*_*i*_ and the information-collection technology is *imperfect*, the true posterior distribution presented in Eq. 13 is a mixture of beta-distributions. There are *n*_*t*_ + 1 possible outcomes of a study with sample size of *n*_*t*_ and so there are *n*_*t*_ + 1 possible values for *j* + *k* in Eq. 13. However, not all the posterior distributions created by updating each of the *m* prior distributions are necessarily unique. Therefore, the true posterior distribution is a mixture of between *m* + *n*_*t*_ and *m* × (*n*_*t*_ + 1) beta-distributions.

Consider the example where *m* = 2, *x*_*t*_ = ((19, 20), (20, 19)), *n*_*t*_ = 5. Again, let $\hat {x}_{t}$ denote the posterior belief state. Given the sample size of *n*_*t*_ = 5 there are 6 possible true outcomes, by which we mean the unobservable number of actual positive samples in the study, which correspond to *n*_*t*_ + *m* = 7 possible unique beta-distributions $\hat {x}_{t}=((19,25), (20,24),(21,23), (22,22),(23,21), (24,$ 20), (25, 19)) which each contribute to the posterior mixture distribution. When we observe a specific number of positives in the sample, the imperfect information-collection technology results in a distribution over the true number of positives in the sample and, therefore, weights on each component in the posterior mixture distribution (given by Eq. 13).

#### A.2.2 Mean and variance of mixtures of beta-distributions

The posterior distribution of $\tilde {p}_{t}$ given sample information *v*_*t*_ collected using an imperfect information-collection technology is a mixture of beta-distributions. In this section, we first derive equations for the mean and variance for a general mixture of beta-distributions (with simplified notation) to show their relationship to the mean, and more generally, to the parameters of the component distributions. Then, we do the appropriate substitutions to present the conditional mean and variance of the posterior distribution *f*_*p*|*v*_.

Consider a distribution *f*_*Y*_(*y*) which is a mixture of *M* beta-distributions where the *i*-th component of the mixture has weight *w*_*i*_, parameters *a*_*i*_ and *b*_*i*_, and mean *μ*_*i*_:

$$f_{Y}(y) \,=\, \sum\limits_{i=1}^{m} w_{i} \frac{\Gamma(a_{i} \,+\, b_{i})}{\Gamma(a_{i}) {\Gamma}(b_{i})} y^{a_{i} -1} (1\,-\,y)^{b_{i} -1} \text{where }\! \sum\limits_{i=1}^{m} w_{i} \,=\, 1. $$

We show that the mean of a mixture of beta-distributions is the weighted mean of each mixture component:

14 $$\begin{array}{@{}rcl@{}} \mathbb{E}[ f_{Y}(y) ] \!&=&\! {{\int}_{0}^{1}} y f_{Y}(y) dy\\ \!&=&\! {{\int}_{0}^{1}} y \left(\sum\limits_{i=1}^{m} w_{i} \frac{\Gamma(a_{i} \,+\, b_{i})}{\Gamma(a_{i}) {\Gamma}(b_{i})} y^{a_{i} -1} (1\,-\,y)^{b_{i} -1} \right) dy \\ \!&= &\! \sum\limits_{i=1}^{m} w_{i} \frac{\Gamma(a_{i} + b_{i})}{\Gamma(a_{i}) {\Gamma}(b_{i})} {{\int}_{0}^{1}} y^{a_{i}} (1-y)^{b_{i} -1} dy\\ \!&=&\! \sum\limits_{i=1}^{m} w_{i} \frac{\Gamma(a_{i} + b_{i})}{\Gamma(a_{i}) {\Gamma}(b_{i})} \frac{\Gamma(a_{i}+1) {\Gamma}(b_{i})}{\Gamma(a_{i} + b_{i} + 1)} \\ \!&=&\! \sum\limits_{i=1}^{m} w_{i} \frac{a_{i}}{a_{i} + b_{i}} = \sum\limits_{i=1}^{m} w_{i} \mu_{i}. \end{array} $$

We also derive the variance of *f*_*Y*_(*y*):

15 $$\begin{array}{@{}rcl@{}} \mathbb{V}[ f_{Y}(y)] \!\!&= &\!\! {{\int}_{0}^{1}} y^{2} f_{Y}(y) dy \,-\, (\mathbb{E}[ f_{Y}(y) ])^{2} \\ \!\!&=&\!\! {{\int}_{0}^{1}} y^{2} \left(\sum\limits_{i=1}^{m} w_{i} \frac{\Gamma(a_{i} \,+\, b_{i})}{\Gamma(a_{i}) {\Gamma}(b_{i})} y^{a_{i} -1} (1\,-\,y)^{b_{i} -1} \right)\\ && \times dy \,-\, \left(\sum\limits_{i=1}^{m} w_{i} \mu_{i} \right)^{2} \\ \!\!&= &\!\! \sum\limits_{i=1}^{m} w_{i} \frac{\Gamma(a_{i} \,+\, b_{i})}{\Gamma(a_{i}) {\Gamma}(b_{i})} {{\int}_{0}^{1}} y^{a_{i} +1} (1\,-\,y)^{b_{i} -1}\\ &&\times dy \,-\, \left(\sum\limits_{i=1}^{m} \omega_{i} \mu_{i} \right)^{2} \\ \!\!&= &\!\! \sum\limits_{i=1}^{m} w_{i} \frac{\Gamma(a_{i} \,+\, b_{i})}{\Gamma(a_{i}) {\Gamma}(b_{i})} \frac{\Gamma(a_{i}\,+\,2) {\Gamma}(b_{i})}{\Gamma(a_{i} \,+\, b_{i}\,+\,2)}\,-\, \left(\sum\limits_{i=1}^{m} \omega_{i} \mu_{i} \right)^{2} \\ \!\!&=&\!\! \sum\limits_{i=1}^{m} w_{i} \frac{(a_{i}\,+\,1)a_{i}} {(a_{i} \,+\, b_{i}\,+\,1)(a_{i} \,+\, b_{i})} \,-\, \left(\sum\limits_{i=1}^{m} w_{i} \mu_{i} \right)^{2}. \end{array} $$

##### **Mean and variance of the posterior distribution***f*_*p*| *v*_

. The posterior distribution of $\tilde {p}_{t}$, *f*_*p*|*v*_, is a mixture of beta-distribution (Proposition 1). Using Eqs. 14 and 15 with appropriate substitutions, we can identify:

16 $$\begin{array}{@{}rcl@{}} \mathbb{E}[f_{p|v}(p | \tilde{v}_{t}=v_{t})] &=& \sum\limits_{j=0}^{v_{t}} \sum\limits_{k=0}^{n_{t} -v_{t}} \sum\limits_{i=1}^{m} \omega^{\prime}_{j, k, i}\\ &&\times\frac{a_{t,i}+j+k}{a_{t,i} + b_{t,i}+n_{t}} \end{array} $$

and

17 $$\begin{array}{@{}rcl@{}} \mathbb{V}[f_{p|v}(p | \tilde{v}_{t}\,=\,v_{t})] \!&=&\! \sum\limits_{j=0}^{v_{t}} \sum\limits_{k=0}^{n_{t} -v_{t}} \sum\limits_{i=1}^{m} \omega^{\prime}_{j, k, i}\\ &&\times\frac{(a_{t,i}\,+\,j\,+\,k\,+\,1)(a_{t,i}\,+\,j\,+\,k)} {(a_{t,i} \,+\, b_{t,i}\,+\,n_{t}\,+\,1)(a_{t,i} \,+\, b_{t,i}\,+\,n_{t})} \\ && - \left(\mathbb{E}[f_{p|v}(p | \tilde{v}_{t}\,=\,v_{t})] \right)^{2} \end{array} $$

where *ω*_*i*_ is the prior weight on the *i*-th component of the prior distribution, and

$$\omega^{\prime}_{j, k, i} = \frac{ \omega_{i} {q_{1}^{j}} (1-q_{2})^{v_{t}-j} (1-q_{1})^{k} q_{2}^{n_{t}-v_{t}-k} \frac{\Gamma(a_{t,i}+b_{t,i}) {\Gamma}(a_{t,i} +j+k) {\Gamma}(b_{t,i} +n_{t}-j -k)} { {\Gamma}(j+1) {\Gamma}(v_{t}-j+1) {\Gamma}(k+1) {\Gamma}(n_{t}-v_{t}-k+1) {\Gamma}(a_{t,i}){\Gamma}(b_{t,i}) {\Gamma}(a_{t,i} + b_{t,i} +n_{t})}} { {\sum}_{r=0}^{v_{t}} {\sum}_{s=0}^{n_{t} -v_{t}} {\sum}_{i=1}^{m} \frac{ \omega_{i} {q_{1}^{r}} (1-q_{2})^{v_{t}-r} (1-q_{1})^{s} q_{2}^{n_{t} - v_{t}-s} {\Gamma}(a_{t,i} + b_{t,i}) {\Gamma}(a_{t,i} +r+s) {\Gamma}(b_{t,i} +n_{t}-r -s)}{\Gamma(r+1) {\Gamma}(v_{t}-r+1) {\Gamma}(s+1) {\Gamma}(n_{t}-v_{t}-s+1){\Gamma}(a_{t,i}) {\Gamma}(b_{t,i}) {\Gamma}(a_{t,i} + b_{t,i} +n_{t})}} . $$

### A.3 Quality of the Posterior Distribution Approximation

We performed extensive numerical simulations to test the accuracy of the approximation. We generated exact posterior distributions under the following conditions:

- Single beta-distribution priors with
  $$\begin{array}{@{}rcl@{}} \mu(x) &\in& \{0.001, 0.0025, 0.005, 0.0075, 0.01, 0.02,\\ &&\quad 0.03, 0.04\} \text{ and }\\ \sigma(x) &\in& \{0.001, 0.002, ..., 0.006\}. \end{array} $$
- Information-collection technologies with
  $$\begin{array}{@{}rcl@{}} q_{1} \!&\in&\! \{0.7, 0.8, 0.9, 0.95, 0.97, 0.99, 1\} \text{ and }\\ q_{2} \!&\in&\! \{0.7, 0.8, 0.9, 0.95, 0.97, 0.99, 0.999, 0.9996, 1\}. \end{array} $$
- Sample sizes *n* ∈{100, 500, 1000, 2500, 5000}.
- Observations *v* at percentiles of
  $$\begin{array}{@{}rcl@{}} \{0.00001, 0.0001, 0.001, 0.002, 0.0025, 0.005, ...,\\ 0.9925, 0.9975, 0.998, 0.999, 0.9999, 0.99999\}. \end{array} $$

In total, 757,781 exact distributions were generated, approximately 600,000 of which had means and variance in the policy-relevant region for our numerical analysis (*μ*(*x*) ∈ (0, 0.04) × *σ*(*x*) ∈ (0, 0.008)). We calculated the Kolmogorov-distance—the maximum distance between the cumulative distribution functions—between each exact beta-mixture posterior distribution and a single beta-distribution with the same mean and standard deviation.

The Kolmogorov-distance between the cumulative density function of the exact posterior distributions and that of the approximation with matching mean and variance was generally small (< 2%) (Fig. 6). The Kolmogorov distances only increased in magnitude for very small means or small means and large standard deviations. Kolmogorov distances above 2% typically appeared only in the stopping region of our numerical example or in the upper left-hand section of the information collection region with small means and high standard deviations (*σ*(*x*) > 0.006). Based on the numerical experiments, for the purposes of informing the practical policy decision, the approximate belief update using moment matching proves to be of reasonably high quality.

### A.4 Derivation of Dynamics in Mean-Variance space

The state *x*_*t*_ = (*a*_*t*_,*b*_*t*_), which contains the parameters of the distribution of $\tilde {p}_{t}$ representing the policy-maker’s current beliefs, follows a law of motion of the form

$$x_{t+1} = \phi(\hat{x}_{t}) = \left[ \begin{array}{cc} z & 0 \\ 1-z & 1 \end{array} \right] \hat{x}_{t} , $$

where *z* ∈ (0, 1) is the decay rate and $\hat {x}_{t}=(\hat {a}_{t}, \hat {b}_{t}) =\psi (x_{t},n_{t},v_{t},q)$ is the Bayesian update of *x*_*t*_ given *v*_*t*_ positive observations out of a test of *n*_*t*_ individuals in the current cohort.

First, we see that

$$x_{t+1} = \left[ \begin{array}{cc} a_{t+1} \\ b_{t+1} \end{array} \right] = \left[ \begin{array}{cc} z \hat{a}_{t} \\ (1-z) \hat{a}_{t} + \hat{b}_{t} \end{array} \right] . $$

Derivation of *μ*(*x*_*t*+1_) beginning with the expectation of a beta-distribution:

$$\begin{array}{@{}rcl@{}} \mu(x_{t+1}) &=& \frac{a_{t+1}} { a_{t+1} + b_{t+1}} = \frac{z \hat{a}_{t}}{z \hat{a}_{t} + (1-z) \hat{a}_{t} + \hat{b}_{t}}\\ &=& \frac{z \hat{a}_{t}}{ \hat{a}_{t} + \hat{b}_{t}} = z \mu(\hat{x}_{t}). \end{array} $$

Derivation of *σ* ^2^(*x*_*t*+1_) beginning with the variance of a beta-distribution:

$$\begin{array}{@{}rcl@{}} \sigma^{2}(x_{t+1}) \!\!&= &\!\! \frac{a_{t+1} b_{t+1}} { (a_{t+1} \,+\, b_{t+1})^{2} (a_{t+1} \,+\, b_{t+1} \,+\, 1 )}\\ \!\!&= &\!\! \frac{(z \hat{a}_{t}) ((1\,-\,z) \hat{a}_{t} \,+\, \hat{b}_{t})} { (z \hat{a}_{t} \,+\, (1-z) \hat{a}_{t} \,+\, \hat{b}_{t} )^{2} (z \hat{a}_{t} \,+\, (1\,-\,z) \hat{a}_{t} \,+\, \hat{b}_{t} \,+\, 1 )}\\ \!\!&=&\!\! \frac{z (1\,-\,z) \hat{a}_{t}^{2} \,+\, z \hat{a}_{t} \hat{b}_{t}} { (\hat{a}_{t} \,+\, \hat{b}_{t} )^{2} (\hat{a}_{t} \,+\, \hat{b}_{t} \,+\, 1 )}\\ \!\!&= &\!\! z (1\,-\,z) \left(\frac{ \hat{a}_{t}^{2}} { (\hat{a}_{t} \,+\, \hat{b}_{t} )^{2} (\hat{a}_{t} \,+\, \hat{b}_{t} \,+\, 1 )} \!\times\! \frac{\hat{b}_{t}}{\hat{b}_{t}} \!\times\! \frac{\hat{a}_{t} \,+\, \hat{b}_{t}}{\hat{a}_{t} \,+\, \hat{b}_{t}}\right)\\ &&\!\!+ z \left(\frac{ \hat{a}_{t} \hat{b}_{t} } { (\hat{a}_{t} \,+\, \hat{b}_{t} )^{2} (\hat{a}_{t} \,+\, \hat{b}_{t} \,+\, 1 )} \right) \end{array} $$

$$\begin{array}{@{}rcl@{}} \quad\quad\quad~~~\!\!&= &\!\! z (1-z) \left(\frac{ \hat{a}_{t} \hat{b}_{t}} { (\hat{a}_{t} + \hat{b}_{t} )^{2} (\hat{a}_{t} + \hat{b}_{t} + 1 )}\right.\\ &&\quad\quad\quad\quad\left.\times \frac{\hat{a}_{t}}{\hat{a}_{t} + \hat{b}_{t}} \times \frac{\hat{a}_{t} + \hat{b}_{t}}{\hat{b}_{t}} \right)\\ &&+ z \sigma^{2}(x_{t}) \\ \!\!&= &\!\! z (1-z) \sigma^{2}(\hat{x}_{t}) \left(\frac{\mu(\hat{x}_{t})}{1-\mu(\hat{x}_{t})} \right) + z \sigma^{2}(\hat{x}_{t}) \\ \!\!&= &\!\! \sigma^{2}(\hat{x}_{t}) \left(z + z (1-z) \left(\frac{\mu(\hat{x}_{t})}{1-\mu(\hat{x}_{t})} \right)\right). \end{array} $$

### A.5 Derivation of Eq. 8

For $\mu (x_{0})\geq \frac {\gamma }{\theta }$, given Eq. 1 and using the fact that *μ*(*x*_*t*_) = *z* ^*t*^ *μ*(*x*_0_), we can identify the optimal time to stop the intervention, *T*(*x*_0_), which is the first period in which the intervention has a nonpositive INMB. Specifically, we seek the value of *t* such that *E*[*g*(*x*_*t*_)] = 0.

$$\begin{array}{@{}rcl@{}} E\left[g(\tilde{p}_{t})\right] = \theta \mu(x_{t}) - \gamma & =& \theta z^{t} \mu(x_{0}) - \gamma = 0 \\ z^{t} & =& \frac{\gamma}{\theta \mu(x_{0})} \\ t & =& \frac{1}{\ln(z)} \ln \left(\frac{\gamma}{\theta \mu(x_{0})} \right) \end{array} $$

Since decisions can only be made at discrete time intervals, we identify the optimal time to stop the intervention, *T*(*x*_0_), as the first integer period in which the intervention has a nonpositive INMB

$$T(x_{0}) = \left\lceil \frac{1}{\ln(z)} \ln \left(\frac{\gamma}{\theta \mu(x_{0})} \right) \right\rceil . $$

### A.6 Proof of Proposition 2

Rewriting Eq. 9 as

$$V_{\text{NoInfo}}(x_{0}) = \sum\limits_{t=0}^{\infty}\delta^{t}(\theta z^{t} \mu(x_{0}) - \gamma ) \mathbf{1}_{\{t\leq T(x_{0})\}}, $$

where *T*(*x*_0_) is the optimal time to stop the intervention, the claim follows from the fact that each term is a nondecreasing, convex function. Specifically for each *t*, *δ* ^*t*^(*𝜃* *z* ^*t*^ *μ*(*x*_0_) − *γ*) is linear in *μ*(*x*_0_) with *𝜃* *δ* ^*t*^ *z* ^*t*^ ≥ 0 and *T*(*x*_0_) nondecreasing in *μ*(*x*_0_). Noting that when *t* = *T*(*x*_0_) = *t*(*x*_0_), (*𝜃* *z* ^*t*^ *μ*(*x*_0_) − *γ*) = 0, we can conclude that *δ* ^*t*^(*𝜃* *z* ^*t*^ *μ*(*x*_0_) − *γ*)**1**_{*t* ≤ *T*(*x*__0_)} is a continuous, nondecreasing convex function.

### A.7 Proof of Proposition 3

We apply Proposition 5 in Smith and McCardle [20], which states that if (a) the current-period reward function satisfies the structural property (such as convexity and monotonicity in *μ*(*x*_*t*_)) for each action, and (b) the state transitions satisfy a stochastic version of the structural property for each action, then the value function satisfies the structural property in a finite-horizon setting. In our setting, which is in principle infinite-horizon up to the stopping time, if the structural property is satisfied in the final period, just before the optimal stopping time, then the previous-period value function is obtained via maximization over functions that each satisfy the structural property. Thus, if the structural property is preserved by maximization, then the previous-period value function also satisfies the structural property. For the proof we assume a perfect detection technology to simplify exposition. We extend to the general case at the end of the proof.

First, condition (a) is satisfied because the current-period expected reward, $\mathbb {E}[g(\tilde {p}_{t}, u_{t})|x_{t}]$, is nondecreasing in *μ*(*x*_*t*_) and *σ* ^2^(*x*_*t*_) and (at least weakly) convex in *μ*(*x*_*t*_) for any action $u_{t}\in \mathcal {D} \times \mathcal {N}$.

Second, the Bayesian update *ψ* preserves stochastic dominance of the beta-distributed prior, in the sense that if one prior is stochastically dominated by another prior, the corresponding posteriors will exhibit the same dominance relationship, under first-order stochastic dominance (FOSD) and second-order stochastic dominance (SOSD).^5^ In particular, an increase of the mean *μ*(*x*_*t*_) will result in an increase of the posterior mean, and an increase of the variance *σ* ^2^(*x*_*t*_) in an increase of the posterior variance. To see this for SOSD, consider a mean preserving spread, so *x* *t*(1), *x* *t*(2) with *μ*(*x* *t*(1)) = *μ*(*x* *t*(2)) and *σ* ^2^(*x* *t*(1)) < *σ* ^2^(*x* *t*(2)). Then *σ* ^2^(*x* *t*(1)) < *σ* ^2^(*x* *t*(2)) implies *a* *t*(1) + *b* *t*(1) > *a* *t*(2) + *b* *t*(2), and $\mathbb {V}[\tilde {s}_{t}| x_{t}^{(1)}, n_{t}] \leqslant \mathbb {V}[\tilde {s}_{t}| x_{t}^{(2)}, n_{t}]$. The conditional variance of the next-period belief given *n*_*t*_ = *η* ≥ 0 samples is $\mathbb {V}[x_{t+1}|x_{t}, \tilde {s}_{t}, n_{t}=\eta ] = \frac {z(a_{t}+\tilde {s}_{t})(a_{t}+b_{t}+\eta -z(a_{t} + \tilde {s}_{t}))}{(a_{t} + b_{t} + \eta )^{2}(a_{t}+b_{t}+\eta +1)}$. The variance of the next-period belief is obtained using the law of total variance, so $\mathbb {V}[x_{t+1}|x_{t}^{(1)}, n_{t}=\eta ] <\mathbb {V}[x_{t+1}|x_{t}^{(2)}, n_{t}=\eta ]$; hence, *ϕ* ∘ *ψ* is increasing in *σ* ^2^. Thus, the Bayesian update is increasing in *μ*(*x*_*t*_) and *σ* ^2^(*x*_*t*_), and the same holds true for its beta-approximation *ψ* introduced in Section 2.2. The state-transition function *ϕ* is linear (time-invariant) in *μ*(*x*_*t*_) and linear (time-variant) in *σ* ^2^(*x*_*t*_), so that the Bayesian-updated state-transition function *ϕ* ∘ *ψ* is increasing in (*μ*(*x*_*t*_),*σ* ^2^(*x*_*t*_)).

Finally, the convexity in *μ*(*x*_*t*_) survives the maximization in the Bellman equation given that the objective function is supermodular in (*u*_*t*_,*μ*(*x*_*t*_)). Since the sum of nondecreasing convex functions is nondecreasing and convex, we only need a terminal condition to satisfy the backwards-induction approach presented by Smith and McCardle [20]. Note that since 0 < *z* < 1, $\lim _{t \to \infty } \mu (\phi (\psi (x_{t}, n_{t}, \tilde {v}_{t}, q))) = 0$ and $\lim _{t \to \infty } \sigma ^{2}(\phi (\psi (x_{t}, n_{t}, \tilde {v}_{t}, q))) = 0$. Therefore, there exists a time $T <\infty $ for which, given any initial state, an optimal policy is to stop the intervention, i.e., *u*_*T*_ = (0, 0), and *V* (*x*_*T*_) = 0. Through the mean- and, ultimately, variance-reducing dynamics, with or without the variance-reducing acquisition of information, any initial state eventually approaches a ‘termination’ state over time. Since the reward of this state is zero, which is nondecreasing and convex, we conclude that *V* (*x*_*t*_) is nondecreasing and convex in *μ*(*x*_*t*_); by a similar argument it is also increasing in *σ* ^2^(*x*_*t*_).

This proof relies mainly on the stochastic-dominance ordering, and it therefore directly extends to the case with misclassification. Condition (a) continues to be satisfied, since it relates only to the current period and is not influenced by information collection. Second, as before, Bayesian updating preserves stochastic dominance of the beta-mixture prior, in the sense that if one prior stochastically dominates another prior, the corresponding posteriors conserve the dominance ordering, for FOSD and SOSD. Finally, imperfect information collection does not affect the supermodularity of the objective function in (*u*_*t*_,*μ*(*x*_*t*_)), so convexity in *μ*(*x*_*t*_) survives the maximization in the Bellman equation. This allows for backward induction starting with the ‘stop intervention’-region at zero reward, as described above.

### A.8 Proof of Corollary 1

For the case where no information is available, this corollary was already shown to be true with the derivation of a threshold policy in Section 3.1. For the general case, with or without information collection in the current or future periods, we rely on the properties of the value function demonstrated in Proposition 3: if *μ*(*x* *t*(1)) < *μ*(*x* *t*(2)) and *σ*(*x* *t*(1)) = *σ*(*x* *t*(2)), then $V(x_{t}^{(1)}) \leqslant V(x_{t}^{(2)})$. This directly implies that, if it is optimal to do the intervention with *μ*(*x* *t*(1)), then it is also optimal to do the intervention at *μ*(*x* *t*(2)). Furthermore, if it is *not* optimal to do the intervention with *μ*(*x* *t*(2)) then, because $V(x_{t}^{(1)}) \leqslant V(x_{t}^{(2)})$, it is also not optimal to do the intervention at *μ*(*x* *t*(1)).

### A.9 Proof of Corollary 2

For the case where no information is available, the optimal policy does not depend on *σ*(*x*_*t*_) (see the threshold policy in Section 3.1). For the general case, with or without information collection in the current or future periods, we rely on the properties of the value function demonstrated in Proposition 3: if *μ*(*x* *t*(1)) = *μ*(*x* *t*(2)) and *σ*(*x* *t*(1)) < *σ*(*x* *t*(2)), then $V(x_{t}^{(1)}) \leqslant V(x_{t}^{(2)})$. This directly implies, if it is optimal to do the intervention with *σ*(*x* *t*(1)), then it is also optimal to do the intervention at *σ*(*x* *t*(2)). Furthermore, if it is *not* optimal to do the intervention with *σ*(*x* *t*(2)) then, because $V(x_{t}^{(1)}) \leqslant V(x_{t}^{(2)})$, it is also not optimal to do the intervention at *σ*(*x* *t*(1)).

### A.10 Proof of Proposition 4

Misclassification results in a posterior distribution which has greater variance than would occur with the same sample size and a perfect detection technology. When evaluating the expected value with information collection, greater variance in the posterior implies an expected value over a larger number of states where the optimal next action is to not do the intervention and, therefore, have a value of 0.

Given a perfect detection technology, smaller sample sizes will have greater variance in the posterior distribution than larger sample sizes. Therefore, misclassification can be thought of as an effective sample size reduction or, equivalently, an increase in cost for each full unit of information.

An increase in cost or a decrease in the expected value of the next state given information that was collected this period, decrease the value of the information-collection alternative. Therefore, there are fewer states for which information collection is the optimal action, i.e., the action providing the greatest expected value. Misclassification has no effect on the option not to do the intervention, and has a limited effect on the immediate option not to collect information this period (it would influence this option only when the optimal action of a subsequent state is to collect information).

### A.11 Supplemental methods and results for HCV screening example

#### A.11.1 Development of linear INMB

A schematic of the HCV screening decision problem for a single cohort is presented in Fig. 7.

We denote *λ* as the willingness-to-pay threshold, *q*_1_ and *q*_2_ as the test sensitivity and specificity, *C*_*S*_ > 0 as the cost of the screening test, $B_{S} \leqslant 0$ as the quality-of-life loss from the screening test, $C_{FP} \geqslant 0$ as the cost of correcting a false-positive test result, $B_{FP} \leqslant 0$ as the quality-of-life loss from a false-positive test result, and we denote the lifetime discounted costs and benefits of the true-positive, false-negative, and true-negative screening outcomes, *C*_1_, *C*_2_, *C*_3_, and *B*_1_, *B*_2_, *B*_3_, respectively.

The net monetary benefit (NB) of the decision not to screen cohort *t* is

18 $$ \text{NB}_{\text{NoScreening}} \,=\, \lambda \left(\tilde{p}_{t} B_{2} \,+\, (1\,-\,\tilde{p}_{t}) B_{3} \right) - \left(\tilde{p}_{t} C_{2} \,+\, (1\!\,-\,\!\tilde{p}_{t}) C_{3} \right). $$

The net monetary benefit (NB) of the decision not to screen cohort *t* is

19 $$\begin{array}{@{}rcl@{}} \text{NB}_{\text{Screening}} \!\!&= &\!\! \lambda \left(\tilde{p}_{t} q_{1} B_{1} \,+\, \tilde{p}_{t} (1\,-\,q_{1}) B_{2} \,+\, (1\,-\,\tilde{p}_{t})\right.\\ &&\quad\left.\!\times\! (1\,-\,q_{2}) (B_{3}\,+\,B_{FP}) \,+\, (1\,-\,\tilde{p}_{t}) q_{2} B_{3} \,+\, B_{S} \right)\\ &&\,-\,\left(\tilde{p}_{t} q_{1} C_{1} \,+\, \tilde{p}_{t} (1\,-\,q_{1}) C_{2} \,+\, (1\,-\,\tilde{p}_{t})\right.\\ &&\quad\!\times\!\! \left.(1\,-\,q_{2}) (C_{3}\,+\,C_{FP}) \,+\, (1\,-\,\!\tilde{p}_{t}) q_{2} C_{3} \,+\, C_{S} \right). \\ \end{array} $$

The INMB of screening compared to the alternative of not screening is computed as the difference between Eqs. 19 and 18:

$$\begin{array}{@{}rcl@{}} \text{INMB}_{\text{Screening}} \!&= &\! \text{NB}_{\text{Screening}} - \text{NB}_{\text{NoScreening}} \\ \!&= &\! \lambda \left(\tilde{p}_{t} q_{1} B_{1} + \tilde{p}_{t} (1-q_{1}) B_{2} + (1-\tilde{p}_{t})\right. \\ &&\quad\left.\times (1-q_{2}) (B_{3}+B_{FP}) + (1-\tilde{p}_{t})\right. \\ &&\quad\left.\times q_{2} B_{3} + B_{S} \right)\\&&- \left(\tilde{p}_{t} q_{1} C_{1} + \tilde{p}_{t} (1-q_{1}) C_{2} + (1-\tilde{p}_{t})\right. \\ &&\quad\left.\times (1-q_{2}) (C_{3}+C_{FP}) + (1-\tilde{p}_{t})\right. \\ &&\quad\left.\times q_{2} C_{3} + C_{S} \right) \\ && - \left(\lambda \left(\tilde{p}_{t} B_{2} + (1-\tilde{p}_{t}) B_{3} \right)\right. \\ &&\quad\left.- \left(\tilde{p}_{t} C_{2} + (1-\tilde{p}_{t}) C_{3} \right) \right)\\ \!&= &\! \lambda \tilde{p}_{t} \left[ q_{1} B_{1} + (1-q_{1}) B_{2} -(1-q_{2})\right. \\ &&\quad\left.\times (B_{3}+B_{FP}) - q_{2} B_{3} -B_{2} + B_{3} \right] \\ && + \lambda \left[ (1-q_{2}) (B_{3}+B_{FP}) + q_{2} B_{3}\right. \\ &&\quad\quad\left.+ B_{S} - B_{3} \right] \\ && - \tilde{p}_{t} \left[ q_{1} C_{1} + (1-q_{1}) C_{2} - (1-q_{2})\right. \\ &&\quad\quad\left.\times (C_{3}+C_{FP}) - q_{2} C_{3} - C_{2} + C_{3} \right] \\ && - (1\,-\,q_{2}) (C_{3}\,+\,C_{FP}) - q_{2} C_{3} - C_{S} + C_{3} \\ \!&= &\! \tilde{p}_{t} \left(q_{1} \left[ \lambda (B_{1} - B_{2} ) - (C_{1} - C_{2}) \right]\right. \\ &&\quad~\left.\times - (1-q_{2}) \left[ \lambda B_{FP} - C_{FP} \right] \right) \\ && + \lambda B_{S} - C_{S} + (1-q_{2}) (\lambda B_{FP} - C_{FP}) \end{array} $$

With terms collected, the INMB of screening at age 50 compared to not screening at time *t* in a cohort with HCV prevalence $\tilde {p}_{t}$ can be written $\text {INMB}_{t}=\theta \tilde {p}_{t} - \gamma $. The marginal INMB of early diagnosis and treatment for an individual with HCV, *𝜃*, and the fixed INMB of screening, *γ*, are

20 $$ \theta= q_{1} \left[ \lambda (B_{1} - B_{2} ) - (C_{1} - C_{2}) \right] - (1-q_{2}) \left[ \lambda B_{FP} - C_{FP} \right] , $$

and

21 $$ \gamma=C_{S} - \lambda B_{S} - (1-q_{2}) (\lambda B_{FP} - C_{FP}). $$

#### A.11.2 Parameter estimation

Consistent with the recommendations of the US Panel on Cost-Effectiveness in Health and Medicine, we adopted a societal perspective, considered costs and benefits over a lifetime horizon for each cohort, and discounted future costs and health benefits at 3% annually [1]. We measured costs in 2010 US dollars and adjusted for inflation using the Consumer Price Index when appropriate [90]. Benefits are measured in quality-adjusted life-years (QALYs). We assumed a mid-range value for society’s maximum willingness to pay of $75,000 per QALY gained [84].

Estimating the lifetime costs and benefits of each HCV screening outcome for cohorts of asymptomatic 50-year old men and women requires a detailed natural history model of HCV. We used the model by Liu et al. [14] to estimate the lifetime costs and benefits of each HCV screening outcome.

We assumed that the cohort size at each period, the number of people who attend a preventive health exam at age 50, is constant over time, *N*, since there is less than 10% variation from the average population size across cohorts currently aged between 25 and 55 years of age [79]. At the beginning of each period *t*, the policy-maker simultaneously decides whether to screen the current cohort for HCV and whether to conduct a study of sample size *n*_*t*_ to better estimate the current prevalence of HCV. Information arrives at the end of the current period and is used, together with the prevalence dynamics, to inform the screening decision at *t* + 1 for the next cohort. We assumed that the cost of sample information, *K*(*n*_*t*_), is affine in the sample size with a fixed cost of $50,000 and variable cost of $100 [83].

We used the National Health and Nutrition Examination Survey (NHANES) to estimate birth-cohort-specific HCV prevalence, HCV-prevalence dynamics, and the proportion of individuals currently unaware of their infection status. Ultimately, we estimated the HCV prevalence for our initial cohorts, men and women born in 1960 who are currently unaware of their infection status, to be 3.1% (95% CI: 2.4-3.8%) and 1.4% (95% CI: 1.0-1.7%), respectively. Restricting the analysis to individuals born between 1956 and 1980 (*n* = 12,607), we identified the rate of prevalence decay to be 0.893 (95% CI: 0.871-0.915) using logistic regression, controlling for race and gender. We present the detailed methods and results of this primary analysis below.

#### A.11.3 National health and nutrition examination survey (NHANES) analysis

##### **Overview**

The National Center for Health Statistics periodically conducts NHANES to compile representative statistics on the health of the US population [88]. Our analysis includes data collected from 1999 through 2010. Participants were chosen according to a stratified multistage algorithm to produce a representative sample of the civilian, non-institutionalized population of all 50 states and the District of Columbia. Only participants at least age 6 years old were eligible for HCV testing because of low blood sample volume in younger children. Birth years for individuals younger than 85 years, for survey years 1999-2006, and for individuals younger than 80 years old, for survey years 2008-2010, were estimated using their age in months and the 6-month window of the survey. Age in months is not provided for older individuals. Beginning with the 2001/2002 survey, participants testing HCV-positive were informed in writing of their test results, and four months later, they received a follow-up telephone questionnaire.

##### **Statistical Analysis**

All statistical analyses were performed in SAS version 9.3 (SAS Institute, Cary, North Carolina) according to National Center for Health Statistics guidelines [86, 87]. We accounted for the complex survey design using the appropriate study design variables, sampling weights, and by using SAS Survey procedures. Logistic regression analysis was used to identify the rate of HCV-prevalence decay over successive birth cohorts for individuals born between 1956 and 1980. Finally, using the follow up survey in HCV-positive individuals, we estimated the proportion of HCV-positive individuals who were unaware of their infection status prior to participation in NHANES.

##### **Results**

Of 51,587 participants of at least age 6 years surveyed between 1999 and 2010, 45,153 gave a blood sample suitable for HCV-antibody testing (final response rate for testing, 87.5%). Restricting analysis to individuals born between years 1956 and 1980 (n=12,607), we identified the rate of prevalence decay to be 0.893 (95% CI: 0.871-0.915) using logistic regression controlling for race and gender (Table 5). Using the regression, the predicted HCV prevalence for men and women born in 1960 are 4.7% (95% CI: 3.8-5.7%) and 2.9% (95% CI: 2.3-3.6%), respectively.

**Table 5:** Logistic regression predicting HCV prevalence for 1956-1980 birth cohorts using NHANES 1999–2010 (*n* = 12,607)

Variable	Estimate	SE	p-value	Odds Ratio (95% CI)
Intercept	217.7	23.85	< .0001	
Birth year	− 0.113	0.012	< .0001	0.893 (0.872 – 0.915)
Race: Black (vs. non-Black)	0.226	0.073	0.0021	1.57 (1.18 – 2.10)
Gender: Female (vs. Male)	− 0.256	0.071	0.0003	0.6 (0.46 – 0.79)

SE – Standard error; CI – Confidence interval

Since the 2001/02 survey, 500 subjects were identified as HCV-positive and contacted for follow-up which included asking if they were previously aware of their HCV-infection status. The response rate to the follow-up questionnaire was 206 (41%). Using logistic regression, we estimated the proportion of men and women who were unaware of their HCV-infection status prior to participating in the NHANES study to be 55% (95% CI: 46-65%) and 39% (95% CI: 30-39%), respectively (Table 6). Because of the small sample size, we did not stratify analysis by birth year; race was excluded from the final regression model because it was not a significant predictor of prior infection-status awareness.

**Table 6:** Logistic regression predicting the proportion of HCV-positive individuals unaware of their infection status using NHANES 1999–2010 (*n* = 206)

Variable	Estimate	SE	p-value	Odds Ratio (95% CI)
Model 1:				
Intercept	0.00022	0.167	0.998	
Race: Black (vs. non-Black)	0.161	0.157	0.304	1.40 (0.75 – 2.55)
Gender: Female (vs. Male)	− 0.332	0.140	0.017	0.51 (0.30 – 0.89)
Model 2 (Final Model):				
Intercept	− 0.111	0.144	0.444	
Gender: Female (vs. Male)	− 0.329	0.140	0.019	0.52 (0.30 – 0.90)

SE – Standard error; CI – Confidence interval

To compute the HCV prevalence among those who are currently unaware of their infection status, we also needed an estimate of the proportion of individuals who are aware of their HCV-negative status, which is unknown. We assumed it to be 15%, consistent with Liu et al. [14]. Using the logistic regression model to predict birth-cohort-specific HCV prevalence and adjusting for the number of individuals who are unaware of their infection status, we estimated the HCV prevalence for our initial cohorts, men and women born in 1960 who are currently unaware of their infection status, is 3.1% (95% CI: 2.4-3.8%) and 1.4% (95% CI: 1.0-1.7%), respectively.

#### A.11.4 Sensitivity analysis

We performed sensitivity analysis to evaluate the robustness of the optimal policy to uncertainty in model inputs (Table 7). We identified that the general conclusions of our analysis are robust to the uncertainty inputs. Specifically, for women, we find that the optimal time to collect information ranges from immediately to 7 years. For men, we find that the optimal time to collect information ranges from 11-21 years with the exception of scenarios in which we considered a low value of *z*. A very low value of *z* implies the prevalence of HCV is rapidly decreasing across birth cohorts. If this is the case, it is optimal to collect additional information immediately.

**Table 7:** Comparison of optimal policies indicated by various analytic approaches

Case	Adjusted Parameter Value	Optimal Policy ^∗^	Increase in expected INMB compared to screening for 5 years ^∗∗^
Males			
Base Case		Sample 4,000 men in 16 years (1976 birth cohort), then identify optimal action	$ 168, 800, 000
Low *𝜃*	*𝜃* = 3000	Sample in 11 years (1971 birth cohort)	$ 51, 410, 000
High *𝜃*	*𝜃* = 10, 000	Sample in 20 years (1980 birth cohort)	$ 390, 240, 000
Low *γ*	*γ* = 22	Sample in 18 years (1978 birth cohort)	$ 197, 990, 000
High *γ*	*γ* = 40	Sample in 13 years (1973 birth cohort)	$ 120, 800, 000
Low *z*	*z* = 0.871	Sample immediately	$ 185, 690, 000
High *z*	*z* = 0.915	Sample in 21 years (1981 birth cohort)	$ 261, 390, 000
High *κ*_*F*_	*κ*_*F*_ = 250, 000	Sample in 16 years (1976 birth cohort)	$ 168, 600, 000
Low *μ*(*x*_0_)	*μ*(*x*_0_) = 0.024	Sample in 13 years (1973 birth cohort)	$ 106, 040, 000
High *μ*(*x*_0_)	*μ*(*x*_0_) = 0.038	Sample in 18 years (1978 birth cohort)	$ 235, 620, 000
Scenario 1	*𝜃* = 8500;	Sample immediately	$ 257, 240, 000
	*γ* = 25;		
	*z* = 0.88		
Scenario 2	*𝜃* = 5500;	Sample in 14 years (1974 birth cohort)	$ 108, 430, 000
	*γ* = 35;		
	*z* = 0.905		
Females			
Base Case		Sample 4,500 women in 3 years (1963 birth cohort), then identify optimal action	$ 7, 110, 000
Low *𝜃*	*𝜃* = 1500	Never initiate screening / Stop screening	$ 63, 330, 000
High *𝜃*	*𝜃* = 6000	Sample in 7 years (1967 birth cohort)	$ 22, 550, 000
Low *γ*	*γ* = 22	Sample in 4 years (1964 birth cohort)	$ 610, 000
High *γ*	*γ* = 40	Sample in 1 years (1961 birth cohort)	$ 46, 040, 000
Low *z*	*z* = 0.871	Sample immediately	$ 14, 570, 000
High *z*	*z* = 0.915	Sample in 3 years (1963 birth cohort)	$ 2, 460, 000
High *κ*_*F*_	*κ*_*F*_ = 250, 000	Sample in 1 years (1961 birth cohort)	$ 6, 900, 000
Low *μ*(*x*_0_)	*μ*(*x*_0_) = 0.024	Sample immediately	$ 29, 320, 000
High *μ*(*x*_0_)	*μ*(*x*_0_) = 0.038	Sample in 4 years (1964 birth cohort)	$ 340, 000
Scenario 1	*𝜃* = 8500;	Sample immediately	$ 7, 090, 000
	*γ* = 25;		
	*z* = 0.88		
Scenario 2	*𝜃* = 5500;	Never initiate screening / Stop screening	$ 69, 020, 000
	*γ* = 35;		
	*z* = 0.905		

^*^ Sample size for men = 4,000; Sample size for women = 4,500

^**^ Expected value of the optimal policy with the parameter change compared to the expected value of the CDC/USPSTF recommendation of screening for 5 years (calculated with the adjusted input parameters)

## Footnotes

1 A beta-distribution with parameters (*a*,*b*) has mean $\mu = \frac {a}{a + b}$ and variance $\sigma ^{2}=\frac {a b}{(a + b)^{2} (a + b + 1)}$. Through direct substitution and rearrangement, it can be shown that a beta-distribution with mean *μ* and variance *σ* ^2^ has parameters $a=\mu \left (\frac {\mu (1-\mu )}{\sigma ^{2}} -1 \right )$ and $b =(1-\mu ) \left (\frac {\mu (1-\mu )}{\sigma ^{2}} -1 \right )$.

2 The posterior distribution is a weighted sum of component beta-distributions; see Eq. 2. The weights of the exact posterior distribution are generated by the convolution of two binomial distributions; see Eq. 13. The convolution of two binomial distributions creates unimodal weights over the ordered set of component beta-distributions in the mixture (ordered in terms of increasing first parameter). For example, consider a prior *x*_*t*_ = (3, 7). Given a sample size *n*_*t*_ = 5, there are 6 possible true outcomes, by which we mean the potentially unobservable number of actual positive samples in the study. These true outcomes correspond to 6 possible unique beta-distributions with parameters (3, 12), (4, 11), (5, 10), (6, 9), (7, 8), and (8, 7) forming the components of the exact posterior distribution. Because the information technology is imperfect, we have a belief over these possible outcomes equal to the distribution of the actual number of positives given a specific number of observed positives. We can compute this distribution using the weights in Eq. 2, where *j* is the number of actual positives among the *v*_*t*_ observed positives, and *k* is the number of actual positives among the *n*_*t*_ − *v*_*t*_ observed negatives. The probability that the actual number of positives in the sample is *W* is determined by summing all the weights for which *j* + *k* = *W*. The unimodality of the weights over the ordered component distributions ensures the unimodality of the posterior distribution. To complete the numerical example, consider the case where there are 3 observed positives, *v*_*t*_ = 3, and *q* = (0.9, 0.85), which results in weights of 0.028, 0.141, 0.346, 0.414, 0.067, 0.004 over the component beta-distributions in the mixture.

3 Weber [65] uses global optimization to consider the general problem of switching between arbitrary streams of expected benefits allowing for multiple switches, which can be viewed as a deterministic equivalent of the multi-armed bandit problem.

4 Our initial cohort is individuals born in 1960. This result can be interpreted as a recommendation for immediate information collection.
